# Chinese herbal medicine for functional dyspepsia: a network meta-analysis of prokinetic-controlled randomised trials

**DOI:** 10.1186/s13020-021-00556-6

**Published:** 2021-12-20

**Authors:** Leonard Ho, Claire C. W. Zhong, Charlene H. L. Wong, Justin C. Y. Wu, Karina K. H. Chan, Irene X. Y. Wu, Ting Hung Leung, Vincent C. H. Chung

**Affiliations:** 1 School of Chinese Medicine, Faculty of Medicine, The Chinese University of Hong Kong, Shatin, Hong Kong; 2 The Jockey Club School of Public Health and Primary Care, Faculty of Medicine, The Chinese University of Hong Kong, Shatin, Hong Kong; 3 Department of Medicine and Therapeutics, Faculty of Medicine, The Chinese University of Hong Kong, Shatin, Hong Kong; 4 United Christian Nethersole Community Health Service, The Chinese University of Hong Kong Chinese Medicine Clinic Cum Training and Research Centre (Tai Po District), Tai Po, Hong Kong; 5 Xiangya School of Public Health, Central South University, Changsha, China

**Keywords:** Medicine, Chinese traditional, Domperidone, Dyspepsia, Systematic review, Network meta-analysis

## Abstract

**Background:**

Prokinetic is the first-line conventional treatment for functional dyspepsia (FD) in Asia despite potential adverse events. Chinese herbal medicine (CHM) may be an effective and safe substitution. This network meta-analysis (NMA) aimed to evaluate the comparative effectiveness of different CHM formulae for FD against prokinetics.

**Methods:**

Seven international and Chinese databases were searched from their inception to July 2020 for randomised controlled trials (RCTs) on CHM versus prokinetics. Data from each RCT were first pooled using random-effect pairwise meta-analyses and illustrated as risk difference (RD) or standardised mean difference (SMD) with 95% confidence interval (CI). Random-effect NMAs were then performed to evaluate the comparative effectiveness of CHM formulae and displayed as RD with 95% CI or SMD with 95% credible interval (CrI). The GRADE partially contextualised framework was applied for NMA result interpretation.

**Results:**

Twenty-six unique CHM formulae were identified from twenty-eight RCTs of mediocre quality. Pairwise meta-analyses indicated that CHM was superior to prokinetics in alleviating global symptoms at 4-week follow-up (pooled RD: 0.14; 95% CI: 0.10–0.19), even after trim and fill adjustment for publication bias. NMAs demonstrated that Modified Zhi Zhu Decoction may have a moderate beneficial effect on alleviating global symptoms at 4-week follow-up (RD: 0.28; 95% CI: − 0.03 to 0.75). Xiao Pi Kuan Wei Decoction may have a large beneficial effect on alleviating postprandial fullness (SMD: − 2.14; 95% CI: − 2.76 to 0.70), early satiety (SMD: − 3.90; 95% CI: − 0.68 to − 0.42), and epigastric pain (SMD: − 1.23; 95% CI: − 1.66 to − 0.29). No serious adverse events were reported.

**Conclusion:**

Modified Zhi Zhu Decoction and Xiao Pi Kuan Wei Decoction may be considered as an alternative for patients unresponsive to prokinetics. Confirmatory head-to-head trials should be conducted to investigate their comparative effectiveness against prokinetics.

**Supplementary Information:**

The online version contains supplementary material available at 10.1186/s13020-021-00556-6.

## Introduction

Functional dyspepsia (FD) is a common gastrointestinal disorder characterised by postprandial fullness, early satiation, epigastric pain, or epigastric burning that is unexplainable by routine investigations [1]. It has a high prevalence of 10–40% among Western countries and a relatively low prevalence of 5–30% among Asian countries, independent of disease definitions [2]. Based on predominant symptoms, FD can be subdivided into diagnostic subtypes of postprandial distress syndrome (PDS, predominant symptoms include postprandial fullness and early satiety) and epigastric pain syndrome (EPS, predominant symptoms include epigastric burning and epigastric pain) [2], with the former subtype being more prevalent in Asia [3].

Current guidelines recommended several conventional treatments for FD. In the 2017 North American clinical guideline [4], proton pump inhibitors (PPIs) are the first-line treatment for both FD subtypes, followed by tricyclic antidepressants (TCAs). PPIs have a relatively high number needed to treat of eleven [5], and their long-term usage is associated with adverse effects such as acute interstitial nephritis, hip fracture, and *Clostridium difficile* infection [6]. TCAs are associated with adverse events like dry mouth, somnolence, constipation, and urinary retention [4], and some patients tend to avoid TCAs due to the perceived stigma of receiving psychiatric therapy [7]. These imply that the first two treatment options may not help a considerable number of FD patients. In the 2012 Asian Consensus Report on Functional Dyspepsia [8], prokinetics are the first- and second-line treatment for the subtype of PDS and EPS, respectively. Given the relatively higher prevalence of PDS in Asian FD populations, prokinetics, such as domperidone and mosapride, are commonly prescribed for FD patients in China [9] and South Korea [10]. However, recommendations for prokinetics are supported only by very low-quality evidence [11], and certain prokinetics are associated with adverse events ranging from dystonia to life-threatening arrhythmia [4, 12].

Failure of first-line conventional treatment in FD management is not uncommon. For instance, despite the wide use of prokinetics, a study in China revealed that nearly a quarter of FD patients were refractory to conventional treatments [13]. Alternative treatment options for these patients are necessary as they are known to have a longer disease duration, more severe symptom burden, more intense health service utilisation, and higher healthcare-related expenditure [13]. In view of current limitations among guideline-recommended treatments, Chinese herbal medicine (CHM) represents a possible complement or alternative option, especially among patients unresponsive to first-line treatments like PPIs and prokinetics. Indeed, herbal medicine is recommended by the Asian clinical guideline as a potential treatment option after failing a course of 8-week conventional therapy regardless of FD subtypes [8].

Herbal medicine constitutes an important component in many healthcare systems, and strategies for promoting the use of herbal medicine have been outlined by the World Health Organization [14]. CHM is a branch of herbal medicine practice widely adopted in China and other Chinese communities. It refers to the natural medicinal ingredients, including plants, animals, and minerals, and their processed products that are prepared and used under the guidance of Traditional Chinese Medicine (TCM) theories [15]. The cost of herbal medicine for FD management is expected to be low, particularly in regions where herbal medicine has been practised as a tradition [14]. For example, the typical cost for a single-day CHM treatment is only USD5.87 in China [16].

Although a clinical guideline based on expert consensus was published in China on the use of CHM for FD management [17], and it is known to be superior to placebo [18], evidence on the comparative effectiveness of CHM relative to prokinetics has not been synthesised in a systematic manner. Also, as the relative performance of different CHM interventions is unclear, specific recommendations cannot be made to inform routine practice. To clarify the potential role of CHM as an alternative to prokinetics, we explored the comparative effectiveness of different CHM interventions against prokinetics via network meta-analysis (NMA) in this systematic review.

## Methods

### Literature search

Seven electronic databases were searched from their inception to July 2020 [Additional file 1: Appendix 1]. Four were Chinese databases: Wanfang Data, China National Knowledge Infrastructure, SinoMed, and Index to Taiwan Periodical Literature System. Three were international databases: MEDLINE via Ovid, EMBASE via Ovid, and Cochrane Central Register of Controlled Trials. Validated search filters with high sensitivity for randomised controlled trials (RCTs) were applied for searching MEDLINE and EMBASE [19, 20].

### Eligibility criteria

Eligible RCTs must meet the criteria for participants, interventions, controls, and outcomes measures described below, with full-text written in English or Chinese. Systematic reviews, clinical recommendations, conference abstracts, or research protocols were excluded.

#### Participants

RCTs that recruited adult patients diagnosed with FD based on any editions of the Rome diagnostic criteria were eligible. No restrictions on FD subtypes were placed.

#### Interventions and comparisons

RCTs comparing orally administered CHM to prokinetics were eligible. Orally administered CHM could be in the form of single herbs, herbal formulae, or proprietary medicine, with components clearly reported. Comparisons between orally administered CHM were also eligible for inclusion. RCTs on cisapride were excluded as it has been withdrawn in most countries due to life-threatening adverse events [8].

#### Outcomes

RCTs must report the primary outcome of global symptom alleviation. Alleviation of postprandial fullness, early satiety, epigastric pain, and epigastric burning were considered as the secondary outcomes. The primary and secondary outcomes were selected according to current expert recommendations on clinical endpoints for FD trials [21].

### Study selection, data extraction, risk of bias assessment, and quality of evidence assessment

Titles, abstracts, and full-texts of all records were screened as per the eligibility criteria after deduplication with EndNote 20. Characteristics and outcome data of eligible studies were then extracted. In classical TCM theories, a diagnostic pattern refers to the summarisation of the cause, nature, and location of the pathological change at a certain stage of disease [14]. It encompasses information on the patient’s clinical signs and symptoms. Considering the significance of diagnostic pattern in TCM, diagnostic pattern(s) of the participants in each included study was extracted, if reported. TCM function(s) of each identified CHM intervention was obtained from the study as well. Risk of bias assessment was performed using the Cochrane Risk-of-Bias Tool for Randomized Trials 2 [22]. Quality of evidence was assessed for pairwise meta-analyses and NMAs using the GRADE (Grading of Recommendations Assessment, Development and Evaluation) approach [23, 24]. These procedures were performed by two reviewers (Ho and Chan) independently, with disagreements resolved through consensus. Persisted disagreements were settled by a third reviewer (Chung).

### Data analyses

#### Pairwise meta-analysis

To synthesise results of head-to-head comparisons between CHM and prokinetics, random effect pairwise meta-analyses were executed using Review Manager 5.3. Results on the alleviation of global symptoms (rated on dichotomous scale) were pooled and expressed as risk difference (RD) and 95% confidence interval (CI). Results on the alleviation of postprandial fullness, early satiety, epigastric pain, and epigastric burning (rated on continuous scale) were pooled and presented as standardised mean difference (SMD) and 95% CI. All outcome results were pooled as per their length of follow-up [25, 26]. Sensitivity analysis was performed for the primary outcome, comparing pooled results between RCTs with some concerns over risk of bias or at low risk of bias, against those at high risk of bias. Publication bias on the primary outcome was assessed via contour-enhanced funnel plots produced by RStudio 1.3.1073. Trim and fill method was adopted to adjust for publication bias detected [26].

The level of heterogeneity among RCTs was measured with *I*^*2*^ statistics, with *I*^*2*^ < 25%, 25–50%, 50% regarded as low-, moderate-, and high-level heterogeneity, respectively [27]. The following minimally clinically important difference (MCID) values were used to facilitate result interpretation: RD of 0.20 between groups for the primary outcome [23]; and SMD of − 0.50 for the secondary outcomes [23].

#### Network meta-analysis

NMA combines direct and indirect evidence across a network of interventions in a single analysis, allowing the ranking of interventions based on relative efficacy [26]. Direct evidence refers to results from head-to-head comparisons of two interventions within RCTs (for example, *X* versus *Y* and *Y* versus *Z*), while indirect evidence is computed via analysing results from comparisons of two interventions via a common comparator (for example, *X* versus *Z* via *Y*) [28].

In this review, random-effect Bayesian NMAs were carried out on RStudio to evaluate the comparative effectiveness of CHM interventions, via specific prokinetics as common comparators. Results on the primary outcome were analysed using binomial likelihood model [29], while results on the secondary outcomes were analysed using normal likelihood model [30]. Dichotomous and continuous outcomes were expressed as RD and risk ratio (RR) with 95% credible interval (CrI) and SMD with 95% CrI, respectively. The ranking of CHM interventions across different outcomes was determined by the probability of specific CHM being at different ranks and expressed using surface under the cumulative ranking curve (SUCRA) [31].

When drawing conclusions, rankings suggested by SUCRA values should be considered together with the effect magnitude of interventions and relevant certainty of evidence [32, 33]. In this review, the GRADE partially contextualised framework was adopted to facilitate the interpretation of NMA results [32]. In this four-step framework, thresholds for small, moderate, and large beneficial effect were first established in accordance with the MCID of different outcomes. For the primary outcome, an RD value of 0.08 represented a small beneficial effect, 0.20 a moderate beneficial effect, and 0.31 a large beneficial effect [34]. Following the method reported in *Newcombe *et al. [35], RDs were computed from relevant RRs and the expected response (*i.e.* baseline risk) of FD prokinetic treatment. The baseline risk used for both domperidone and mosapride was 0.42, which was extracted from a meta-analysis on prokinetic response [11], with a 95% CI of 0.38 to 0.46 and of 0.38 to 0.47, respectively, computed using the Wilson score method [36]. For the secondary outcomes, an SMD of − 0.20 represented a small beneficial effect, − 0.50 a moderate beneficial effect, and − 0.80 a large beneficial effect [26].

Secondly, for each outcome, different CHM interventions were categorised into “trivial to no beneficial effect”, “small but important beneficial effect”, “moderate beneficial effect”, or “large beneficial effect” based on point estimates of their relative efficacy against specific prokinetics. Thirdly, the interventions were stratified according to the certainty of evidence supporting their relative efficacy which was graded using the GRADE NMA rating system [23, 24]. Lastly, the consistency between the point estimate and ranking of each intervention was evaluated to finalise the classification of all interventions.

## Results

### Study selection and characteristics

**Fig. 1:**
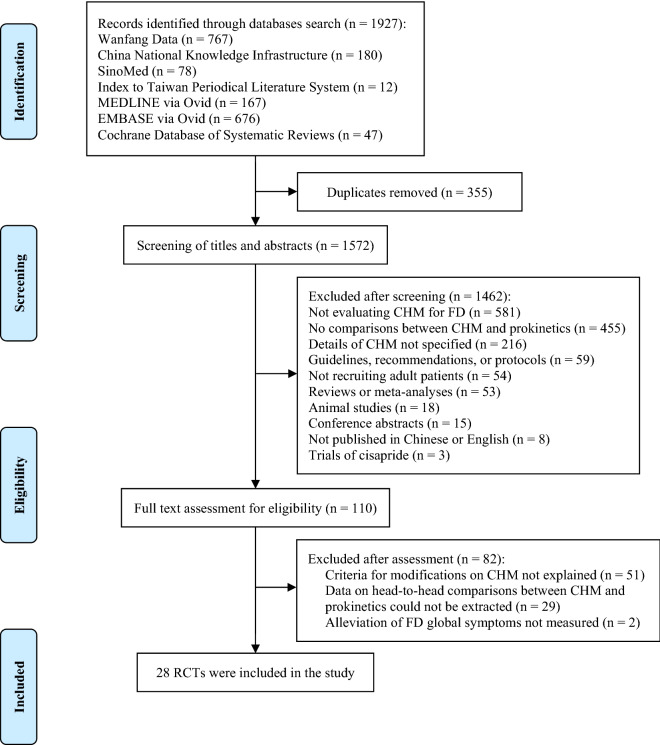
Flow of literature search and selection. *CHM* Chinese herbal medicine, *FD* functional dyspepsia, *RCT* randomised controlled trial

A total of 1,927 citations were yielded from the literature search. 1,572 titles and abstracts were screened after deduplication. 110 potential full-texts then proceeded to further eligibility assessment. Finally, twenty-eight RCTs were included in this study. Selection process is illustrated in Fig. 1.

All RCTs were conducted in China, with only one [37] of them published in English, and spanned 2004 to 2019 (Table 1). A total of 2,736 participants took part in the twenty-eight trials, with an average sample size of 98 (range, 56–202). Average age of the participants ranged from 24.0 to 54.6 years. Duration of their FD symptoms ranged from less than one year to over twenty-four years. Participants in twenty-three trials [37–59] were diagnosed by the Rome III criteria, while those in the other five trials [60–64] were diagnosed by the Rome II criteria. Fifteen out of twenty-eight RCTs adopted TCM diagnostic pattern as part of their inclusion criteria (Table 1). Nine unique TCM diagnostic patterns were included: food stagnation [38], liver qi invading the stomach [40, 43], liver depression and spleen deficiency [49, 61, 62], cold-heat complex [42, 46, 64], liver qi depression [44], spleen deficiency and qi stagnation [46, 51, 54], spleen-stomach weakness [48], spleen qi deficiency [56], and spleen deficiency and dampness-heat [57].

**Table 1:** Characteristics of included studies

Reference (Country)	CHM formula	Prokinetic	Number of participants R/A	Average age (SD)	FD diagnostic criteria (TCM diagnostic pattern(s), if reported)	Duration of FD symptoms	Treatment duration (Length of follow-up)	Outcome
Gong [38] (China)	Liu Wei An Xiao Capsule	Domperidone	CHM group: 82/82 Prokinetic group: 74/74	*Not reported*	Rome III criteria (Food stagnation)	*Not reported*	4 weeks (4 weeks)	Alleviation of global symptoms
Li [60] (China)	Liu Wei An Xiao Capsule	Domperidone	CHM group: 50/50 Prokinetic group: 50/50	CHM group: 41.3 Prokinetic group: 38.6	Rome II criteria	CHM group: 3.8 years in average Prokinetic group: 3.2 years in average	4 weeks (4 weeks)	Alleviation of global symptoms
Zhou [39] (China)	He Wei Decoction	Domperidone	CHM group: 48/48 Prokinetic group: 48/48	CHM group: 37.4 (4.3) Prokinetic group: 36.5 (3.8)	Rome III criteria	*Not reported*	4 weeks (4 weeks)	Alleviation of global symptoms Alleviation of postprandial fullness Alleviation of early satiety Alleviation of epigastric pain
Leng [40] (China)	He Wei Decoction	Domperidone	CHM group: 41/41 Prokinetic group: 39/39	CHM group: 42.0 (13.0) Prokinetic group: 38.6 (14.2)	Rome III criteria (Liver qi invading the stomach)	*Not reported*	4 weeks (4 weeks)	Alleviation of global symptoms Alleviation of postprandial fullness Alleviation of early satiety Alleviation of epigastric pain
Gao [41] (China)	Modified He Gan Decoction	Domperidone	CHM group: 40/40 Prokinetic group: 40/40	CHM group: 41.6 Prokinetic group: 40.1	Rome III criteria	CHM group: 6.1 years in average Prokinetic group: 6.3 years in average	4 weeks (4 weeks)	Alleviation of global symptoms Alleviation of postprandial fullness
Liu [61] (China)	Fu An Decoction	Domperidone	CHM group: 40/40 Prokinetic group: 40/40	CHM group: 31.6 (13.1) Prokinetic group: 32.7 (11.3)	Rome II criteria (Liver depression and spleen deficiency)	CHM group: 4.8 ± 1.7 years Prokinetic group: 4.8 ± 1.4 years	4 weeks (4 weeks)	Alleviation of global symptoms
Lai [42] (China)	Xiao Pi Tong Jiang Decoction	Domperidone	CHM group: 30/30 Prokinetic group: 30/30	CHM group: 37.4 (9.8) Prokinetic group: 39.7 (8.9)	Rome III criteria (Cold-heat complex)	CHM group: 3.6 ± 1.9 years Prokinetic group: 3.4 ± 1.5 years	4 weeks (4 weeks)	Alleviation of global symptoms Alleviation of postprandial fullness Alleviation of early satiety Alleviation of epigastric pain Alleviation of epigastric burning
Cai [43] (China)	Xiao Pi Kuan Wei Decoction	Domperidone	CHM group: 47/47 Prokinetic group: 47/47	CHM group: 35.6 Prokinetic group: 35.9	Rome III criteria (Liver qi invading the stomach)	CHM group: 4.4 ± 0.6 years Prokinetic group: 4.8 ± 0.9 years	4 weeks (4 weeks)	Alleviation of global symptoms Alleviation of postprandial fullness Alleviation of early satiety Alleviation of epigastric pain Alleviation of epigastric burning
Dong [44] (China)	Cai Zhu Jie Yu Decoction	Domperidone	CHM group: 32/32 Prokinetic group: 32/32	CHM group: 44.8 (9.5) Prokinetic group: 45.2 (9.8)	Rome III criteria (Liver qi depression)	CHM group: 3.7 ± 0.9 years Prokinetic group: 3.5 ± 0.7 years	4 weeks (4 weeks)	Alleviation of global symptoms
Liu [45] (China)	Zhi Zhu Kuan Zhong Capsule	Domperidone	CHM group: 97/97 Prokinetic group: 105/105	*Not reported*	Rome III criteria	*Not reported*	4 weeks (4 weeks)	Alleviation of global symptoms
Liu [46] (China)	Xiao Pi Decoction	Domperidone	CHM group: 40/40 Prokinetic group: 40/40	*Not reported*	Rome III criteria (Spleen deficiency and qi stagnation; cold-heat complex)	All participants: 0.3–6.0 years	4 weeks (4 weeks)	Alleviation of global symptoms
Ma [47] (China)	He Wei Xiao Pi Decoction	Domperidone	CHM group: 66/66 Prokinetic group: 60/60	CHM group: 35.8 Prokinetic group: 37.1	Rome III criteria	*Not reported*	4 weeks (4 weeks)	Alleviation of global symptoms
Duan [48] (China)	Modified Zhi Zhu Decoction	Domperidone	CHM group: 60/60 Prokinetic group: 60/60	*Not reported*	Rome III criteria (Spleen-stomach weakness)	CHM group: 0.5–4.5 years Prokinetic group: 0.5–4.5 years	4 weeks (4 weeks)	Alleviation of global symptoms
Wang [49] (China)	Shu Gan Jian Pi He Wei Decoction	Domperidone	CHM group: 50/50 Prokinetic group: 50/50	CHM group: 41.5 (13.4) Prokinetic group: 42.6 (11.6)	Rome III criteria (Liver depression and spleen deficiency)	CHM group: 2.7 ± 1.0 years Prokinetic group: 2.8 ± 1.2 years	4 weeks (4 weeks)	Alleviation of global symptoms
Li [50] (China)	Tiao He Gan Pi Xing Qi Decoction	Domperidone	CHM group: 72/72 Prokinetic group: 56/56	All participants: 46.1 (6.7)	Rome III criteria	*Not reported*	4 weeks (4 weeks)	Alleviation of global symptoms
Huang [51] (China)	Xiang Su Li Qi Decoction	Domperidone	CHM group: 30/30 Prokinetic group: 30/30	CHM group: 38.9 (6.4) Prokinetic group: 39.5 (6.6)	Rome III criteria (Spleen deficiency and qi stagnation)	CHM group: 2.4 ± 0.8 years Prokinetic group: 2.1 ± 0.9 years	4 weeks (4 weeks)	Alleviation of global symptoms
Sheng [52] (China)	Jian Pi Yi Qi Decoction	Domperidone	CHM group: 41/41 Prokinetic group: 41/41	*Not reported*	Rome III criteria	CHM group: 0.5–3.8 years Prokinetic group: 0.5–3.4 years	4 weeks (4 weeks)	Alleviation of global symptoms
Zhao [53] (China)	Tiao Zhong Xiao Pi Decoction	Domperidone	CHM group: 60/60 Prokinetic group: 60/60	CHM group: 25.0 (1.2) Prokinetic group: 24.0 (1.5)	Rome III criteria	CHM group: 2.1 ± 0.6 years Prokinetic group: 2.0 ± 0.4 years	2 weeks (2 weeks)	Alleviation of global symptoms Alleviation of postprandial fullness Alleviation of early satiety Alleviation of epigastric pain Alleviation of epigastric burning
Liu [54] (China)	Wu Mo Decoction	Domperidone	CHM group: 40/40 Prokinetic group: 40/40	CHM group: 45.3 (5.3) Prokinetic group: 46.9 (5.1)	Rome III criteria (Spleen deficiency and qi stagnation)	CHM group: 2.1 ± 0.6 years Prokinetic group: 2.2 ± 0.4 years	2 weeks (2 weeks)	Alleviation of global symptoms
Ma [55] (China)	Cai Hu Shu Gan Powder	Domperidone	CHM group: 30/30 Prokinetic group: 30/30	CHM group: 42.3 (2.1) Prokinetic group: 41.3 (2.2)	Rome III criteria	CHM group: 5.2 ± 3.7 years Prokinetic group: 4.8 ± 3.1 years	2 weeks (2 weeks)	Alleviation of global symptoms
Liu [56] (China)	Wei Kang Ping Decoction	Domperidone	CHM group: 40/40 Prokinetic group: 40/40	CHM group: 54.6 Prokinetic group: 53.8	Rome II criteria (Liver depression and spleen deficiency)	CHM group: 3.5 ± 1.3 years Prokinetic group: 3.4 ± 1.8 years	2 weeks (2 weeks)	Alleviation of global symptoms Alleviation of postprandial fullness
Wang [57] (China)	Qi Zhi Wei Tong Granules	Domperidone	CHM group: 58/58 Prokinetic group: 54/54	All participants: 43.2 (12.1)	Rome II criteria	*Not reported*	2 weeks (2 weeks)	Alleviation of global symptoms
Hu [64] (China)	Ban Xia Xie Xin Decoction	Domperidone	CHM group: 30/30 Prokinetic group: 30/30	CHM group: 37.2 (10.5) Prokinetic group: 39.3 (9.2)	Rome II criteria (Cold-heat complex)	CHM group: 19.2 ± 12.1 years Prokinetic group: 17.3 ± 11.4 years	2 weeks (2 weeks)	Alleviation of global symptoms
Chen [66] (China)	Bu Gan Decoction	Mosapride	CHM group: 28/28 Prokinetic group: 28/28	CHM group: 38.1 Prokinetic group: 37.8	Rome III criteria (Spleen qi deficiency)	CHM group: 2.1 years in average Prokinetic group: 2.2 years in average	4 weeks (4 weeks)	Alleviation of global symptoms Alleviation of postprandial fullness
Wang [57] (China)	Tiao Wei Xiao Pi Decoction	Mosapride	CHM group: 33/33 Prokinetic group: 31/31	CHM group: 47.9 (12.0) Prokinetic group: 44.8 (12.1)	Rome III criteria (Spleen deficiency and dampness-heat)	CHM group: 7.0 ± 5.0 years Prokinetic group: 6.5 ± 5.0 years	4 weeks (4 weeks)	Alleviation of global symptoms Alleviation of postprandial fullness
Huang [58] (China)	Da Li Tong Granules	Mosapride	CHM group: 57/57 Prokinetic group: 57/57	*Not reported*	Rome III criteria	All participants: 0.5–24.0 years	4 weeks (4 weeks)	Alleviation of global symptoms
Zheng [59] (China)	Modified Yue Ju Decoction	Mosapride	CHM group: 60/60 Prokinetic group: 60/60	*Not reported*	Rome III criteria	*Not reported*	6 weeks (6 weeks)	Alleviation of global symptoms
Liu [60] (China)	Xiao Pi II	Mosapride	CHM group: 90/90 Prokinetic group: 90/90	CHM group: 42.0 (15.0) Prokinetic group: 43.0 (14.0)	Rome III criteria	*Not reported*	2 weeks (2 weeks)	Alleviation of global symptoms Alleviation of postprandial fullness Alleviation of epigastric pain

*A* Analysed, *CHM* Chinese herbal medicine, *FD* functional dyspepsia, *R* Recruited, *SD* Standard deviation, *TCM* Traditional Chinese medicine

Twenty-six unique CHM formulae were studied in the twenty-eight RCTs (Table 2). TCM function(s) of the CHM formulae corresponded to the diagnostic pattern(s) adopted in the fifteen RCTs according to TCM theories. Twenty-one formulae were compared against domperidone in twenty-three RCTs, with Liu Wei An Xiao Capsule [38, 60] and He Wei Decoction [39, 40] studied in two RCTs respectively. Five formulae were compared against mosapride in five RCTs [37, 56–59]. Treatment duration ranged from two to six weeks. Twenty trials [38–52, 56–58, 60, 61] had a length of follow-up of four weeks, while seven [37, 53–55, 62–64] and one [59] trials had a 2-week follow-up and 6-week follow-up, respectively.

**Table 2:** Characteristics of included interventions

Reference	CHM formula (dosage, frequency)	TCM function(s) of CHM formula	Ingredients of CHM formula	Prokinetics drugs (dosage, frequency)	Adverse event
Gong [38]	Liu Wei An Xiao Capsule (0.5 g powder per capsule, four capsules each time, three times per day)	Fortifying the spleen and harmonising the stomach Promoting digestion and removing food stagnation	Radix Inulae, Radix et Rhizoma Rhei, Fructus Chebulae, Rhizoma Kaempferiae, Gypsum Rubrum, and Tronae	Domperidone (10 mg per tablet, one tablet each time, three times per day)	CHM group: 8 cases of frequent bowel movements Prokinetic group: 9 cases, including mild diarrhoea (n = 5), mild headache (n = 2), and insomnia (n = 2)
Li [60]	Liu Wei An Xiao Capsule (0.5 g powder per capsule, four capsules each time, three times per day)			Domperidone (10 mg per tablet, one tablet each time, three times per day)	No adverse events reported
Zhou [39]	He Wei Decoction (150 ml decoction, twice per day)	Soothing the liver and harmonising the stomach	Radix Codonopsis, Radix Bupleuri, Fructus Aurantii Immaturus, Cortex Magnoliae Officinalis, Radix et Rhizoma Glycyrrhizae, Pericarpium Citri Reticulatae, Fructus Amomi Rotundus, and Radix Aucklandiae	Domperidone (10 mg per tablet, one tablet each time, three times per day)	No adverse events reported
Leng [40]	He Wei Decoction (Weight in granule not reported, three times per day)			Domperidone (10 mg per tablet, one tablet each time, three times per day)	CHM group: 3 cases, including oral ulcer (n = 2) and diarrhoea (n = 1) Prokinetic group: 1 case of frequent bowel movements
Gao [41]	Modified He Gan Decoction (150 ml decoction, once per day)	Soothing the liver and fortifying the spleen	Radix Angelicae Sinensis, Radix Paeoniae Alba, Radix Codonopsis, Rhizoma Atractylodis Macrocephalae, Poria, Radix Bupleuri, Herba Menthae, Caulis Perillae, Rhizoma Cyperi, Rhizoma Zingiberis Recens, Fructus Jujubae, Radix et Rhizoma Glycyrrhizae, Pericarpium Citri Reticulatae, Massa Medicata Fermentata, and Fructus Amomi	Domperidone (10 mg per tablet, one tablet each time, three times per day)	No adverse events reported
Liu [61]	Fu An Decoction (100 ml decoction, twice per day)	Soothing the liver and fortifying the spleen	Radix Bupleuri, Radix Codonopsis, Radix Paeoniae Alba, Fructus Citri Sarcodactylis, Rhizoma Corydalis. Fructus Toosendan, Radix et Rhizoma Salviae Miltiorrhizae, Rhizoma Atractylodis Macrocephalae, Poria, Radix Aucklandiae, Pericarpium Citri Reticulata, and Radix et Rhizoma Glycyrrhizae	Domperidone (10 mg per tablet, one tablet each time, three times per day)	CHM group: 1 case of mild diarrhoea Prokinetic group: 4 cases, including diarrhoea (n = 2), skin rash (n = 1), and mild headache (n = 1)
Lai [42]	Xiao Pi Tong Jiang Decoction (150 ml decoction, twice per day)	Regulating cold and heat	Pinelliae Rhizoma, Radix Scutellariae, Rhizoma Zingiberis, Radix Codonopsis, Radix Curcumae, Cortex Magnoliae Officinalis, Radix Paeoniae Alba, Radix Bupleuri, and Fructus Aurantii	Domperidone (10 mg per tablet, one tablet each time, three times per day)	No adverse events reported
Cai [43]	Xiao Pi Kuan Wei Decoction (200 ml decoction, twice per day)	Soothing the liver and regulating qi	Radix Bupleuri, Massa Medicata Fermentata, Rhizoma Atractylodis Macrocephalae, Flos Inulae, Pericarpium Citri Reticulatae, Rhizoma Pinelliae, Fructus Aurantii Immaturus, Rhizoma Corydalis, and Radix et Rhizoma Glycyrrhizae	Domperidone (10 mg per tablet, one tablet each time, three times per day)	No adverse events reported
Dong [44]	Cai Zhu Jie Yu Decoction (Volume of decoction not reported, twice per day)	Soothing the liver and resolving qi stagnation	Radix Bupleuri, Rhizoma Atractylodis Macrocephalae, Pericarpium Citri Reticulatae Viride, Fructus Aurantii, Fructus Citri Sarcodactylis, Radix Chuanxiong, Massa Medicata Fermentata, Fructus Amomi, Radix Paeoniae Alba, and Radix et Rhizoma Glycyrrhizae	Domperidone (10 mg per tablet, one tablet each time, three times per day)	No adverse events reported
Liu [45]	Zhi Zhu Kuan Zhong Capsule (0.43 g powder per capsule, three capsules each time, three times per day)	Soothing the liver and fortifying the spleen	Rhizoma Atractylodis Macrocephalae, Fructus Gardeniae, Radix Bupleuri, and Fructus Crataegi	Domperidone (10 mg per tablet, one tablet each time, three times per day)	No adverse events reported
Liu [46]	Xiao Pi Decoction (200 ml decoction, twice per day)	Promoting digestion and removing food stagnation Regulating cold and heat	Fructus Aurantii Immaturus, Poria, Radix Codonopsis, Cortex Magnoliae Officinalis, Rhizoma Coptidis, Pinelliae Rhizoma, Fructus Aurantii, Rhizoma Atractylodis Macrocephalae, Radix et Rhizoma Glycyrrhizae, Radix Aucklandiae, Herba Pogostemonis, Radix et Rhizoma Nardostachyos, Fructus Chebulae, and Fructus Hordei Germinatus	Domperidone (10 mg per tablet, one tablet each time, three times per day)	No adverse events reported
Ma [47]	He Wei Xiao Pi Decoction (100 ml decoction, twice per day)	Fortifying the spleen and harmonising the stomach Promoting digestion and removing food stagnation	Radix Codonopsis, Poria, Rhizoma Atractylodis Macrocephalae, Fructus Aurantii, Radix Paeoniae Alba, Rhizoma Corydalis, Fructus Toosendan, Rhizoma Cimicifugae, Radix Platycodonis, Herba Pogostemonis, Herba Eupatorii, and Radix et Rhizoma Glycyrrhizae	Domperidone (10 mg per tablet, one tablet each time, three times per day)	No adverse events reported
Duan [48]	Modified Zhi Zhu Decoction (Volume of decoction not reported, once per day)	Fortifying the spleen and tonifying qi Promoting digestion and removing food stagnation	Fructus Aurantii Immaturus, Fructus Citri Sarcodactylis, Rhizoma Atractylodis Macrocephalae, Rhizoma Dioscoreae, Semen Nelumbinis, Pericarpium Citri Reticulatae, Endothelium Corneum Gigeriae Galli, Rhizoma Cimicifugae, and Radix et Rhizoma Glycyrrhizae	Domperidone (10 mg per tablet, one tablet each time, three times per day)	No adverse events reported
Wang [49]	Shu Gan Jian Pi He Wei Decoction (200 ml decoction, twice per day)	Soothing the liver and regulating qi Fortifying the spleen and harmonising the stomach	Radix et Rhizoma Salviae Miltiorrhizae, Poria, Rhizoma Atractylodis Macrocephalae, Semen Coicis, Rhizoma Corydalis, Radix Bupleuri, Fructus Aurantii, Radix Paeoniae Alba, Rhizoma Cyperi, Pericarpium Citri Reticulatae, Rhizoma Pinelliae, Fructus Hordei Germinatus, Fructus Crataegi, Massa Medicata Fermentata, Endothelium Corneum Gigeriae Galli, Radix Codonopsis, and Radix et Rhizoma Glycyrrhizae	Domperidone (10 mg per tablet, one tablet each time, three times per day)	No adverse events reported
Li, 2014	Tiao He Gan Pi Xing Qi Decoction (150 ml decoction, twice per day)	Soothing the liver and spleen Moving qi at the middle energizer	Rhizoma Atractylodis Macrocephalae, Fructus Aurantii, Radix Bupleuri, Radix Curcumae, and Radix et Rhizoma Glycyrrhizae	Domperidone (10 mg per tablet, one tablet each time, three times per day)	No adverse events reported
Huang [51]	Xiang Su Li Qi Decoction (150 ml decoction, twice per day)	Warming the spleen and stomach Moving qi at the middle energizer	Radix Astragali, Rhizoma Cyperi, Caulis Perillae, Rhizoma Atractylodis Macrocephalae, Fructus Aurantii, Radix Paeoniae Alba, Radix et Rhizoma Salviae Miltiorrhizae, Radix Aucklandiae, Fructus Amomi, Radix Codonopsis, Poria, and Radix et Rhizoma Glycyrrhizae	Domperidone (10 mg per tablet, one tablet each time, three times per day)	No adverse events reported
Sheng [52]	Jian Pi Yi Qi Decoction (100 ml decoction, twice per day)	Fortifying the spleen and tonifying qi	Radix Codonopsis, Rhizoma Cyperi, Poria, Fructus Jujubae, Radix et Rhizoma Glycyrrhizae, Radix Astragali, Rhizoma Pinelliae, Cortex Magnoliae Officinalis, Fructus Aurantii, Caulis Perillae, and Ramulus Cinnamomi	Domperidone (10 mg per tablet, one tablet each time, three times per day)	CHM group: no adverse events reported Prokinetic group: 5 cases of mild headache
Zhao [53]	Tiao Zhong Xiao Pi Decoction (Volume of decoction not reported, twice per day)	Soothing the liver and regulating qi Fortifying the spleen and harmonising the stomach Promoting digestion and removing food stagnation	Rhizoma Atractylodis Macrocephalae, Fructus Aurantii Immaturus, Radix Bupleuri, Radix Paeoniae Alba, Rhizoma Pinelliae, Rhizoma Coptidis, Fructus Citri Sarcodactylis, Bulbus Lilii, Herba Taraxaci, Fructus Setariae Germinatus, Semen Oroxyli, Flos Rosae Rugosae, Cortex Albiziae, and Radix et Rhizoma Glycyrrhizae	Domperidone (10 mg per tablet, one tablet each time, three times per day)	No adverse events reported
Liu [54]	Wu Mo Decoction	Soothing the liver and regulating qi Fortifying the spleen Promoting digestion and removing food stagnation	Radix Linderae, Lignum Aquilariae Resinatum, Radix Aucklandiae, Semen Arecae, and Radix Codonopsis	Domperidone (10 mg per tablet, one tablet each time, three times per day)	CHM group: 2 cases, including diarrhoea (n = 1) and dizziness (n = 1) Prokinetic group: 2 cases, including urticaria (n = 1) and dizziness (n = 1)
Ma [55]	Cai Hu Shu Gan Powder	Soothing the liver and resolving qi stagnation Moving qi to relieve pain	Pericarpium Citri Reticulatae, Radix Bupleuri, Radix Chuanxiong, Rhizoma Cyperi, Fructus Aurantii, Radix Paeoniae Alba, and Radix et Rhizoma Glycyrrhizae	Domperidone (10 mg per tablet, one tablet each time, three times per day)	No adverse events reported
Liu [62]	Wei Kang Ping Decoction	Soothing the liver and fortifying the spleen	Radix Bupleuri, Fructus Aurantii, Radix Codonopsis, Rhizoma Dioscoreae, Poria, Pericarpium Citri Reticulatae, Rhizoma Acori Tatarinowii, Rhizoma Atractylodis Macrocephalae, Radix Scutellariae, Radix Curcumae, Rhizoma Pinelliae, Radix Aucklandiae, Fructus Hordei Germinatus, Fructus Crataegi, and Massa Medicata Fermentata	Domperidone (10 mg per tablet, one tablet each time, three times per day)	No adverse events reported
Wang [63]	Qi Zhi Wei Tong Granules	Soothing the liver and regulating qi Harmonising the stomach to relieve pain	Radix Bupleuri, Rhizoma Corydalis, Fructus Aurantii, Rhizoma Cyperi, Radix Paeoniae Alba, and Radix et Rhizoma Glycyrrhizae	Domperidone (10 mg per tablet, one tablet each time, three times per day)	No adverse events reported
Hu [64]	Ban Xia Xie Xin Decoction	Regulating cold and heat	Rhizoma Pinelliae, Radix Scutellariae, Rhizoma Coptidis, Radix Codonopsis, Rhizoma Zingiberis, Cortex Magnoliae Officinalis, Fructus Amomi, Radix Paeoniae Alba, Pericarpium Citri Reticulatae, Fructus Jujubae, and Rhizoma Atractylodis Macrocephalae	Domperidone (10 mg per tablet, one tablet each time, three times per day)	No adverse events reported
Chen [56]	Bu Gan Decoction	Fortifying the spleen and tonifying qi Promoting digestion and removing food stagnation	Radix Astragali, Ramulus Cinnamomi, Rhizoma Atractylodis Macrocephalae, Pericarpium Citri Reticulatae, Radix et Rhizoma Asari, Poria, Fructus Amomi, Radix Saposhnikoviae, Rhizoma Pinelliae, Endothelium Corneum Gigeriae Galli, Fructus Hordei Germinatus, Cortex Magnoliae Officinalis, and Radix et Rhizoma Glycyrrhizae	Mosapride (5 mg per tablet, one tablet each time, three times per day)	No adverse events reported
Wang [57]	Tiao Wei Xiao Pi Decoction	Soothing the liver and regulating qi Fortifying the spleen Clearing dampness-heat	Radix Codonopsis, Poria, Rhizoma Atractylodis Macrocephalae, Rhizoma Corydalis, Fructus Toosendan, Herba Taraxaci, Semen Coicis, Caulis Bambusae in Taenia, Fructus Aurantii, Caulis Perillae, Rhizoma Pinelliae, and Radix et Rhizoma Glycyrrhizae	Mosapride (5 mg per tablet, one tablet each time, three times per day)	No adverse events reported
Huang [58]	Da Li Tong Granules	Clearing heat and resolving qi stagnation Harmonising the stomach and directing qi downward Promoting digestion and removing food stagnation	Radix Bupleuri, Radix Aucklandiae, Rhizoma Pinelliae, Fructus Crataegi, Caulis Sinomenii, Rhizoma Corydalis, Fructus Aurantii Immaturus, Pericarpium Citri Reticulatae, Herba Taraxaci, Semen Arecae, Radix Codonopsis, and Massa Medicata Fermentata	Mosapride (5 mg per tablet, one tablet each time, three times per day)	CHM group: no adverse events reported Prokinetic group: 1 case of mild diarrhoea
Zheng [59]	Modified Yue Ju Decoction	Soothing the liver and regulating qi Fortifying the spleen and harmonising the stomach Promoting digestion and removing food stagnation	Rhizoma Cyperi, Radix Aucklandiae, Fructus Aurantii, Cortex Magnoliae Officinalis, Pericarpium Citri Reticulatae, Radix Chuanxiong, Rhizoma Atractylodis, Fructus Gardeniae, and Massa Medicata Fermentata	Mosapride (5 mg per tablet, one tablet each time, three times per day)	No adverse events reported
Liu [37]	Xiao Pi II	Fortifying the spleen and harmonising the stomach Promoting digestion and removing food stagnation	Radix Codonopsis, Rhizoma Atractylodis Macrocephalae, Rhizoma Atractylodis, Poria, Rhizoma Pinelliae, Fructus Amomi, Pericarpium Citri Reticulatae, Fructus Aurantii, and Radix et Rhizoma Glycyrrhizae	Mosapride (5 mg per tablet, one tablet each time, three times per day)	No adverse events reported

*CHM* Chinese herbal medicine, *TCM* Traditional Chinese medicine

### Risk of bias assessment

The overall risk of bias among the included studies was mediocre, with none of them being at low risk, twenty-four having some concerns, and four being at high risk [Additional file 1: Appendix 2]. Those having some concerns [37, 39–44, 46, 48–59, 61–64] did not implement blinding for trial participants, carers, and people delivering interventions. Moreover, they did not report details on allocation sequence generation or provide information on whether the data were analysed following a pre-specified analysis plan. For those at high risk of bias [38, 45, 47, 60], despite limitations described, they did not report details on baseline differences groups.

### Pairwise meta-analysis

**Fig. 2:**
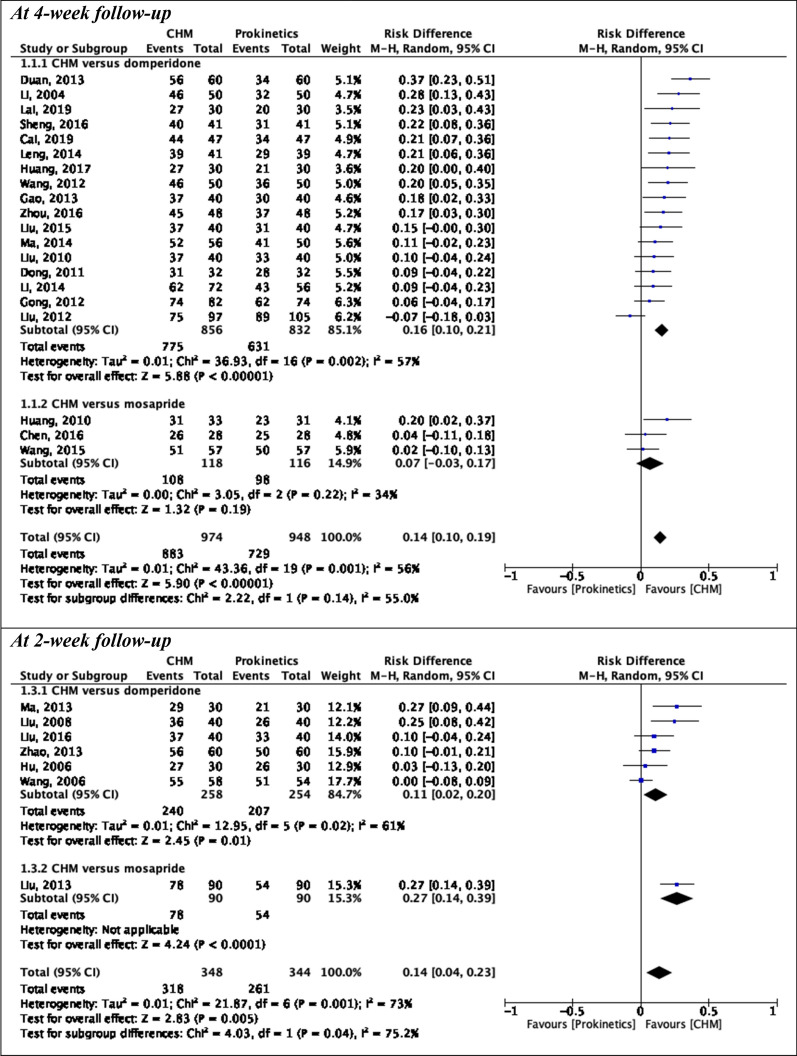
Pairwise meta-analyses on alleviation of global symptoms at different follow-up periods: Chinese herbal medicine versus prokinetics. *CHM* Chinese herbal medicine, *CI* Confidence interval, *SD* Standard deviation

**Fig. 3:**
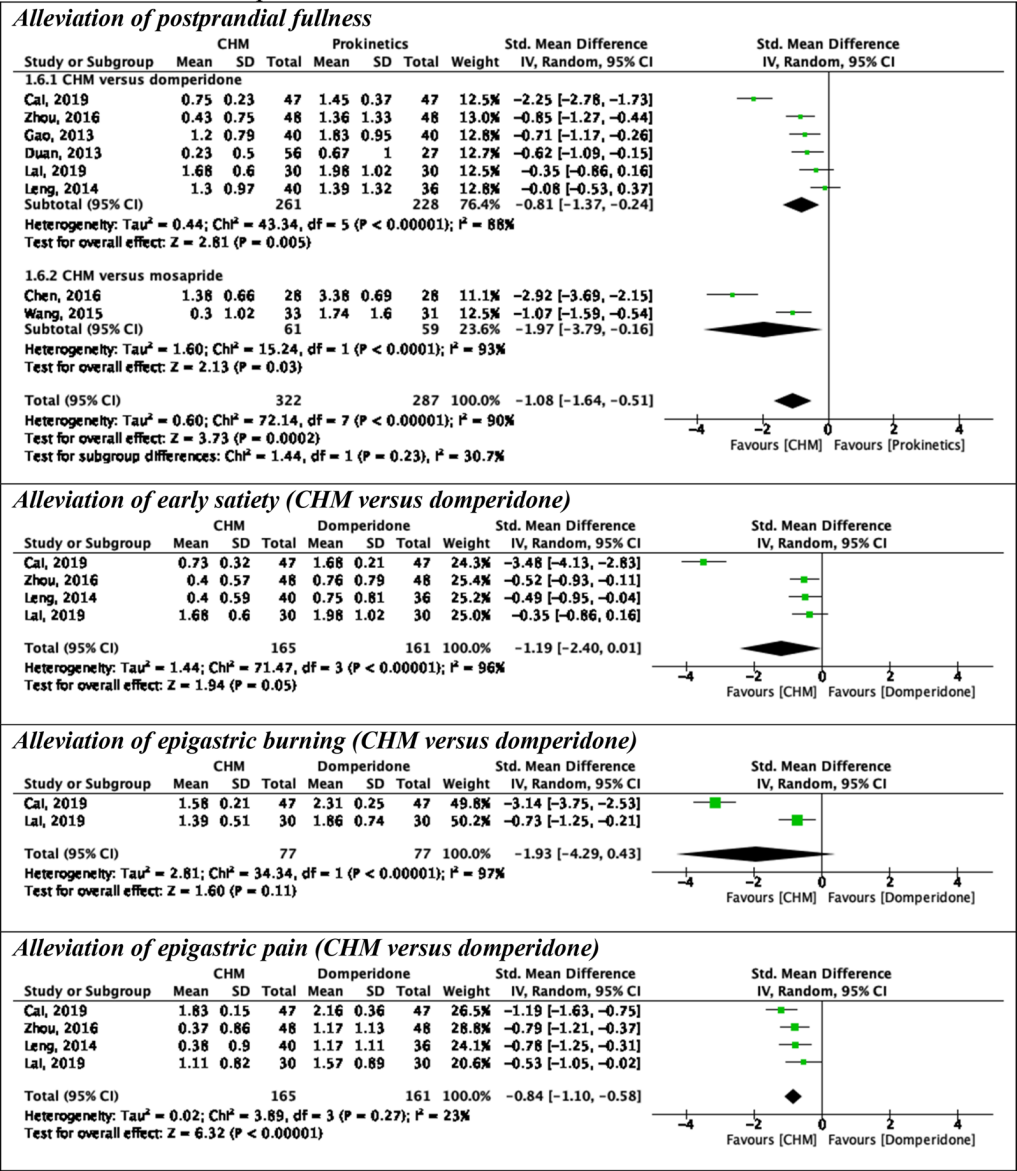
Pairwise meta-analyses on secondary outcomes at 4-week follow-up: Chinese herbal medicine versus prokinetics. *CHM* Chinese herbal medicine, *CI* Confidence interval, *SD* Standard deviation.

Six pairwise meta-analyses were conducted to compare CHM with prokinetics in alleviating global symptoms, postprandial fullness, early satiety, and epigastric pain (Figs. 2, 3).

#### Alleviation of global symptoms

Compared to prokinetics, CHM had a stronger effect in alleviating global symptoms at 4-week follow-up (20 RCTs; pooled RD: 0.14; 95% CI: 0.10–0.19; p < 0.00001; *I*^*2*^ = 56%; low-quality of evidence) (Table 3). CHM was also superior to domperidone alone (17 RCTs; pooled RD: 0.16; 95% CI: 0.10–0.21; p < 0.00001; *I*^*2*^ = 57%; low-quality of evidence). Substantial heterogeneity was observed in both results. No significant difference was found between CHM and mosapride (3 RCTs; pooled RD: 0.07; 95% CI: − 0.03 to 0.17; p = 0.19; *I*^*2*^ = 34%; low-quality of evidence). At 2-week follow-up, CHM was more effective than prokinetics (7 RCTs; pooled RD: 0.14; 95% CI: 0.04–0.23; p = 0.005; *I*^*2*^ = 73%; moderate-quality of evidence) and domperidone alone (6 RCTs; pooled RD: 0.11; 95% CI: 0.02–0.20; p = 0.01; *I*^*2*^ = 61%; moderate-quality of evidence). High-level heterogeneity existed in both pooling results. The MCID of 0.20 RD was not met by any comparisons above.

**Table 3:** Effect estimates and quality of evidence ratings for comparisons in pairwise meta-analyses on alleviation of global symptoms

Outcome	Study design (Number of participants)	Risk of bias	Inconsistency	Indirectness	Imprecision	Publication bias	Pooled result (95% CI)	Quality
Alleviation of global symptoms (4-week follow-up)	20 RCTs (1924 participants)	No serious	No serious	No serious	Serious	Strongly suspected	RD: 0.14 (0.10, 0.19) Trim and fill adjusted RD: 0.10 (0.05, 0.15) RR: 1.21 (1.11, 1.25)	⨁⨁◯◯ Low
Alleviation of global symptoms (4-week follow-up) *(Included only RCTs on CHM versus domperidone)*	17 RCTs (1688 participants)	No serious	No serious	No serious	Serious	Strongly suspected	RD: 0.16 (0.10, 0.21) Trim and fill adjusted RD: 0.12 (0.06, 0.17) RR: 1.20 (1.13, 1.28)	⨁⨁◯◯ Low
Alleviation of global symptoms (4-week follow-up) *(Included only RCTs on CHM versus mosapride)*	3 RCTs (234 participants)	No serious	No serious	No serious	Very serious	Not applicable	RD: 0.07 (− 0.03, 0.17) RR: 1.08 (0.96, 1.21)	⨁⨁◯◯ Low
Alleviation of global symptoms (2-week follow-up)	7 RCTs (692 participants)	No serious	No serious	No serious	Serious	Not applicable	RD: 0.14 (0.04, 0.23) RR: 1.18 (1.04, 1.35)	⨁⨁⨁◯ Moderate
Alleviation of global symptoms (2-week follow-up) *(Included only RCTs on CHM versus domperidone)*	6 RCTs (512 participants)	No serious	No serious	No serious	Serious	Not applicable	RD: 0.11 (0.02, 0.20) RR: 1.13 (1.01, 1.26)	⨁⨁⨁◯ Moderate

*CHM* Chinese herbal medicine, *CI* Confidence interval, *RCT* Randomised controlled trial, *RD* risk difference, *RR* risk ratio

#### Alleviation of postprandial fullness

When compared with prokinetics, CHM showed a stronger effect in alleviating postprandial fullness at 4-week follow-up (8 RCTs; pooled SMD: − 1.08; 95% CI: − 1.64 to − 0.51; p = 0.0002; *I*^*2*^ = 90%; moderate-quality of evidence) (Table 4), with the effect size exceeded the MCID of − 0.50 SMD. CHM was also superior to domperidone (6 RCTs; pooled SMD: − 0.81; 95% CI: − 1.37, − 0.24; p = 0.005; *I*^*2*^ = 88%; low-quality of evidence) and mosapride alone (6 RCTs; pooled SMD: − 1.97; 95% CI: − 3.79, − 0.16; p = 0.03; *I*^*2*^ = 93%; very low-quality of evidence). Substantial heterogeneity was detected in all results.

**Table 4:** Effect estimates and quality of evidence ratings for comparisons in pairwise meta-analyses on secondary outcomes at 4-week follow-up

Outcome	Study design (Number of participants)	Risk of bias	Inconsistency	Indirectness	Imprecision	Publication bias	Pooled result (95% CI)	Quality
Alleviation of postprandial fullness	8 RCTs (609 participants)	No serious	Serious	No serious	No serious	Not applicable	SMD: − 1.08 (− 1.64, − 0.51)	⨁⨁⨁◯ Moderate
Alleviation of postprandial fullness *(Included only RCTs on CHM versus domperidone)*	6 RCTs (489 participants)	No serious	Serious	No serious	Serious	Not applicable	SMD: − 0.81 (− 1.37, − 0.24)	⨁⨁◯◯ Low
Alleviation of postprandial fullness *(Included only RCTs on CHM versus mosapride)*	2 RCTs (120 participants)	No serious	Serious	No serious	Very serious	Not applicable	SMD: − 1.97 (− 3.79, − 0.16)	⨁◯◯◯ Very low
Alleviation of early satiety	4 RCTs (326 participants)	No serious	Serious	No serious	Very serious	Not applicable	SMD: − 1.19 (− 2.40, 0.10)	⨁◯◯◯ Very low
Alleviation of epigastric burning	2 RCTs (154 participants)	No serious	Serious	No serious	Very serious	Not applicable	SMD: − 1.93 (− 4.29, 0.43)	⨁◯◯◯ Very low
Alleviation of epigastric pain	4 RCTs (326 participants)	No serious	No serious	No serious	Very serious	Not applicable	SMD: − 0.84 (− 1.10, − 0.58)	⨁⨁◯◯ Low

A negative SMD indicated an effect favouring Chinese herbal medicine, while a positive SMD indicated an effect favouring prokinetics

*CI* Confidence interval, *RCT* Randomised controlled trial, *SMD* Standardised mean difference

**Table 5:** Comparative effectiveness of different Chinese herbal medicines: domperidone as comparator

Alleviation of global symptoms at 4-week follow-up (shown in RD and 95% CI)															
MZZD															
0.13 (− 0.19, 0.69)	XTD														
0.14 (− 0.17, 0.70)	0.04 (− 0.22, 0.52)	XKD													
0.14 (− 0.17, 0.71)	0.04 (− 0.22, 0.52)	0.03 (− 0.22, 0.46)	JYD												
0.15 (− 0.17, 0.72)	0.05 (− 0.22, 0.53)	0.03 (− 0.22, 0.48)	0.03 (− 0.22, 0.48)	SJHD											
0.15 (− 0.17, 0.74)	0.05 (− 0.22, 0.54)	0.03 (− 0.22, 0.49)	0.03 (− 0.22, 0.48)	0.03 (− 0.23, 0.48)	XSLD										
0.16 (− 0.14, 0.64)	0.05 (− 0.19, 0.47)	0.04 (− 0.20, 0.41)	0.03 (− 0.20, 0.41)	0.03 (− 0.20, 0.40)	0.03 (− 0.20, 0.43)	HWD									
0.17 (− 0.16, 0.76)	0.07 (− 0.21, 0.57)	0.05 (− 0.21, 0.52)	0.04 (− 0.21, 0.51)	0.04 (− 0.22, 0.50)	0.04 (− 0.22, 0.52)	0.02 (− 0.20, 0.40)	MHGD								
0.16 (− 0.13, 0.64)	0.06 (− 0.19, 0.48)	0.04 (− 0.19, 0.42)	0.04 (− 0.19, 0.42)	0.04 (− 0.20, 0.42)	0.04 (− 0.20, 0.44)	0.02 (− 0.20, 0.40)	0.02 (− 0.20, 0.39)	LAXC							
0.19 (− 0.15, 0.80)	0.08 (− 0.20, 0.59)	0.06 (− 0.21, 0.54)	0.06 (− 0.21, 0.53)	0.06 (− 0.21, 0.53)	0.06 (− 0.21, 0.55)	0.04 (− 0.19, 0.42)	0.04 (− 0.22, 0.50)	0.03 (− 0.20, 0.41)	XPD						
0.22 (− 0.13, 0.85)	0.10 (− 0.19, 0.63)	0.08 (− 0.19, 0.58)	0.08 (− 0.20, 0.57)	0.08 (− 0.20, 0.56)	0.08 (− 0.20, 0.59)	0.06 (− 0.18, 0.45)	0.06 (− 0.20, 0.53)	0.05 (− 0.18, 0.45)	0.05 (− 0.21, 0.51)	HXD					
0.22 (− 0.13, 0.87)	0.11 (− 0.19, 0.65)	0.09 (− 0.19, 0.59)	0.09 (− 0.20, 0.59)	0.08 (− 0.20, 0.58)	0.09 (− 0.20, 0.60)	0.06 (− 0.18, 0.46)	0.07 (− 0.20, 0.55)	0.05 (− 0.18, 0.46)	0.05 (− 0.21, 0.52)	0.03 (− 0.22, 0.46)	TGXD				
0.22 (− 0.13, 0.87)	0.11 (− 0.19, 0.66)	0.09 (− 0.19, 0.60)	0.09 (− 0.19, 0.59)	0.08 (− 0.20, 0.58)	0.09 (− 0.20, 0.61)	0.06 (− 0.18, 0.47)	0.07 (− 0.20, 0.55)	0.06 (− 0.18, 0.46)	0.05 (− 0.21, 0.52)	0.03 (− 0.22, 0.47)	0.03 (− 0.22, 0.46)	FAD			
0.23 (− 0.13, 0.88)	0.12 (− 0.19, 0.67)	0.10 (− 0.19, 0.61)	0.10 (− 0.19, 0.60)	0.09 (− 0.19, 0.59)	0.10 (− 0.19, 0.62)	0.07 (− 0.17, 0.48)	0.08 (− 0.20, 0.56)	0.06 (− 0.18, 0.47)	0.06 (− 0.21, 0.53)	0.04 (− 0.21, 0.48)	0.03 (− 0.22, 0.48)	0.03 (− 0.22, 0.47)	CZJD		
0.28 (− 0.03, 0.75)	0.15 (− 0.11, 0.57)	0.13 (− 0.11, 0.50)	0.13 (− 0.11, 0.49)	0.12 (− 0.11, 0.49)	0.13 (− 0.11, 0.52)	0.10 (− 0.07, 0.34)	0.11 (− 0.12, 0.46)	0.09 (− 0.07, 0.34)	0.09 (− 0.13, 0.44)	0.07 (− 0.14, 0.38)	0.06 (− 0.14, 0.38)	0.06 (− 0.14, 0.38)	0.06 (− 0.15, 0.36)	DOMP	
0.36 (− 0.07, > 1.00)	0.23 (− 0.13, 0.89)	0.20 (− 0.14, 0.81)	0.20 (− 0.14, 0.81)	0.20 (− 0.14, 0.79)	0.20 (− 0.15, 0.83)	0.17 (− 0.12, 0.66)	0.17 (− 0.15, 0.76)	0.16 (− 0.12, 0.64)	0.16 (− 0.16, 0.72)	0.13 (− 0.17, 0.65)	0.12 (− 0.17, 0.65)	0.13 (− 0.17, 0.65)	0.12 (− 0.18, 0.63)	0.05 (− 0.15, 0.36)	ZZKC

Alleviation of global symptoms at 2-week follow-up (shown in RD and 95% CI)						
CHSP						
0.02 (− 0.20, 0.39)	WKPD					
0.11 (− 0.15, 0.53)	0.11 (− 0.15, 0.53)	TXD				
0.12 (− 0.15, 0.54)	0.11 (− 0.15, 0.54)	0.02 (− 0.19, 0.34)	WMD			
0.16 (− 0.13, 0.63)	0.16 (− 0.13, 0.62)	0.05 (− 0.18, 0.41)	0.05 (− 0.18, 0.41)	BXD		
0.17 (− 0.11, 0.63)	0.17 (− 0.12, 0.63)	0.06 (− 0.16, 0.41)	0.06 (− 0.17, 0.42)	0.03 (− 0.18, 0.37)	QWG	
0.16 (− 0.06, 0.50)	0.16 (− 0.07, 0.49)	0.06 (− 0.11, 0.29)	0.06 (− 0.12, 0.3)	0.02 (− 0.15, 0.26)	0.01 (− 0.14, 0.22)	DOMP

Alleviation of postprandial fullness at 4-week follow-up (shown in SMD and 95% CrI)					
XKD					
− 1.76 (− 2.52, 0.37)	MHGD				
− 2.03 (− 3.01, 2.88)	– 0.04 (− 0.94, 2.53)	MZZD			
− 2.12 (− 2.99, 1.52)	− 0.27 (− 1.48, 1.55)	− 0.54 (− 1.37, 1.21)	HWD		
− 2.09 (− 3.51, 1.25)	− 0.24 (− 2.92, 1.6)	0.31 (− 5.25, 1.45)	− 0.28 (− 3.94, 2.10)	XTD	
− 2.14 (− 2.76, 0.70)	− 0.39 (− 1.55, 0.54)	− 0.64 (− 2.18, 0.74)	− 0.12 (− 1.68, 0.80)	− 0.34 (− 2.28, 3.30)	DOMP

Alleviation of early satiety at 4-week follow-up (shown in SMD and 95% CrI)			
XKD			
− 3.27 (− 4.81, 1.95)	HWD		
− 3.27 (− 6.34, 0.24)	− 0.49 (− 4.38, 1.84)	XTD	
− **3.90** **(**− **4.68, **− **0.42)**	− 0.58 (− 2.69, 2.06)	− 0.04 (− 2.12, 1.46)	DOMP

Alleviation of epigastric pain at 4-week follow-up (shown in SMD and 95% CrI)			
XKD			
− 0.53 (− 1.3, 0.65)	HWD		
− 0.23 (− 1.14, 0.36)	0.01 (− 0.77, 1.03)	XTD	
− **1.23** **(**− **1.66, **− **0.29)**	− **0.64** **(**− **1.18, **− **0.33)**	− **0.70** **(**− **1.44, **− **0.35)**	DOMP

*BXD* Ban Xia Xie Xin decoction, *CHSP* Cai Hu Shu Gan powder, *CI* Confidence interval, *CrI* Credible interval, *CZJD* Cai Zhu Jie Yu decoction, *DOMP* Domperidone, *FAD* Fu An decoction, *HWD* He Wei decoction, *HXD* He Wei Xiao Pi decoction, *TGXD* Tiao He Gan Pi Xing Qi decoction, *TXD* Tiao Zhong Xiao Pi decoction, *JYD* Jian Pi Yi Qi decoction, *LAXC* Liu Wei An Xiao capsule, *MHGD* Modified He Gan decoction, *MZZD* Modified Zhi Zhu decoction, *QWG* Qi Zhi Wei Tong granules, *RD* Risk difference, *SJHD* Shu Gan Jian Pi He Wei decoction, *SMD* Standardised mean difference, *WKPD* Wei Kang Ping decoction, *WMD* Wu Mo decoction, *XKD* Xiao Pi Kuan Wei decoction, *XPD* Xiao Pi decoction, *XSLD* Xiang Su Li Qi decoction, *XTD* Xiao Pi Tong Jiang decoction, *ZZKC* Zhi Zhu Kuan Zhong capsule

#### Alleviation of early satiety

There was no significant difference between CHM and domperidone in alleviating early satiety at 4-week follow-up (4 RCTs; pooled SMD: − 1.19; 95% CI: − 2.40 to 0.10; p = 0.05; *I*^*2*^ = 96%; very low-quality of evidence) (Table 4). High-level heterogeneity was observed in this comparison.

#### Alleviation of epigastric burning

No significant difference was found between CHM and domperidone in alleviating epigastric burning at 4-week follow-up (4 RCTs; pooled SMD: − 1.93; 95% CI: − 4.29 to 0.43; p = 0.11; *I*^*2*^ = 97%; very low-quality of evidence) (Table 4). High-level heterogeneity existed for this meta-analysis.

#### Alleviation of epigastric pain

CHM was more effective than domperidone in alleviating epigastric pain at 4-week follow-up (4 RCTs; pooled SMD: − 0.84; 95% CI: − 1.10 to − 0.58; p < 0.00001; *I*^*2*^ = 23%; low-quality of evidence), with moderate-level heterogeneity (Table 4). The effect size was higher than the MCID of − 0.50 SMD.

### Sensitivity analysis

**Fig. 4:**
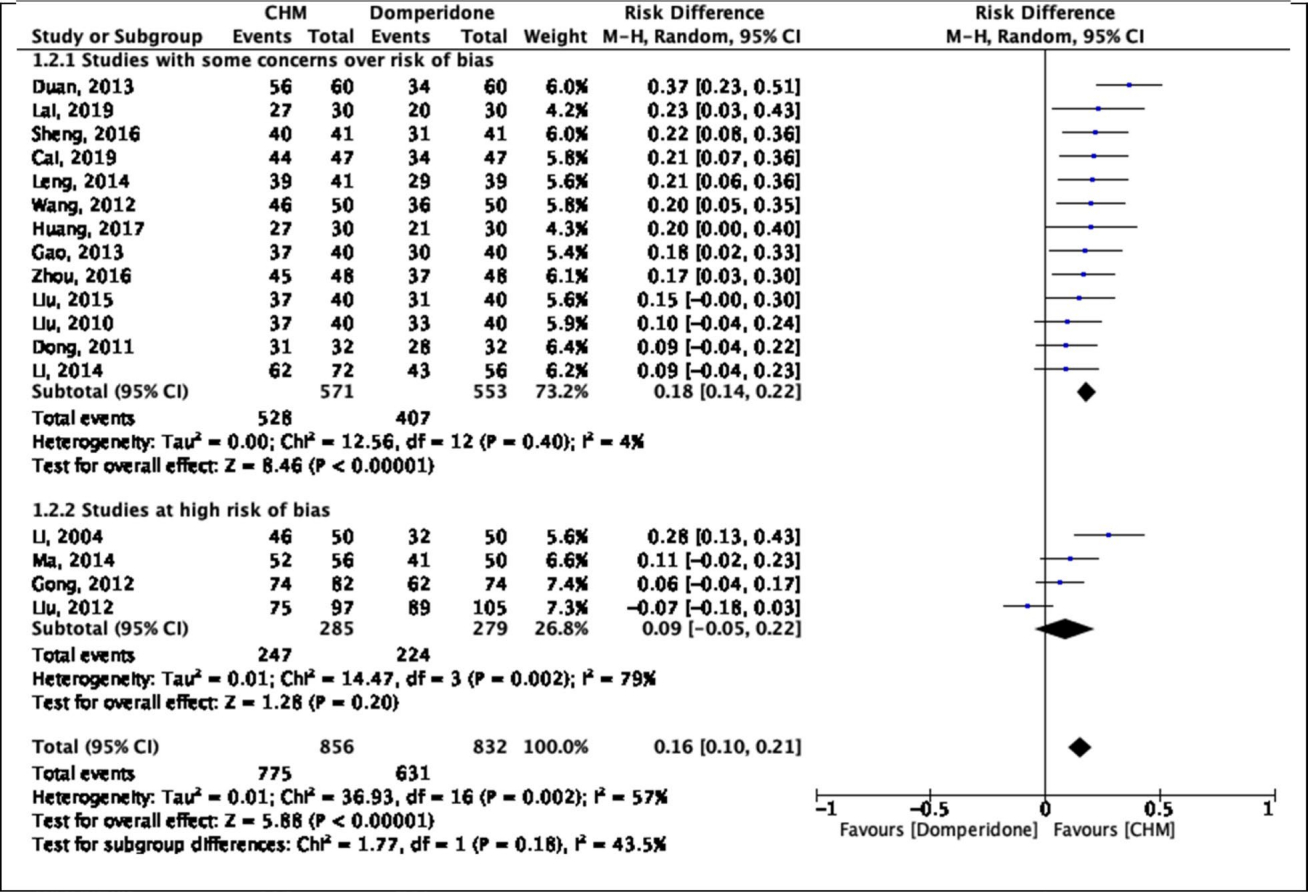
Pairwise meta-analyses on alleviation of global symptoms at 4-week follow-up: Chinese herbal medicine versus domperidone—Subgroup analysis. *CHM* Chinese herbal medicine, *CI* Confidence interval

Sensitivity analysis comparing pooled results from “studies with some concerns over risk of bias” and “studies at high risk of bias” is illustrated in Fig. 4. There was no significant subgroup difference (p = 0.18) between the two groups, implying that the difference in risk of bias level did not influence the pooled results on global symptom alleviation at 4-week.

### Publication bias assessment

Judging from visual inspection of contour-enhanced funnel plots [Additional file 1: Appendix 3a–3b], evidence of funnel plot asymmetry was observed, indicating the potential presence of publication bias favouring CHM in alleviating global symptoms at 4-week follow-up, as compared to all prokinetics and domperidone alone. After applying the trim and fill adjustment, CHM remained to be superior to prokinetics (adjusted RD: 0.10; 95% CI: 0.05–0.15) and domperidone alone (adjusted RD: 0.12; 95% CI: 0.06–0.17) (Table 3).

### Network meta-analysis

**Fig. 5:**
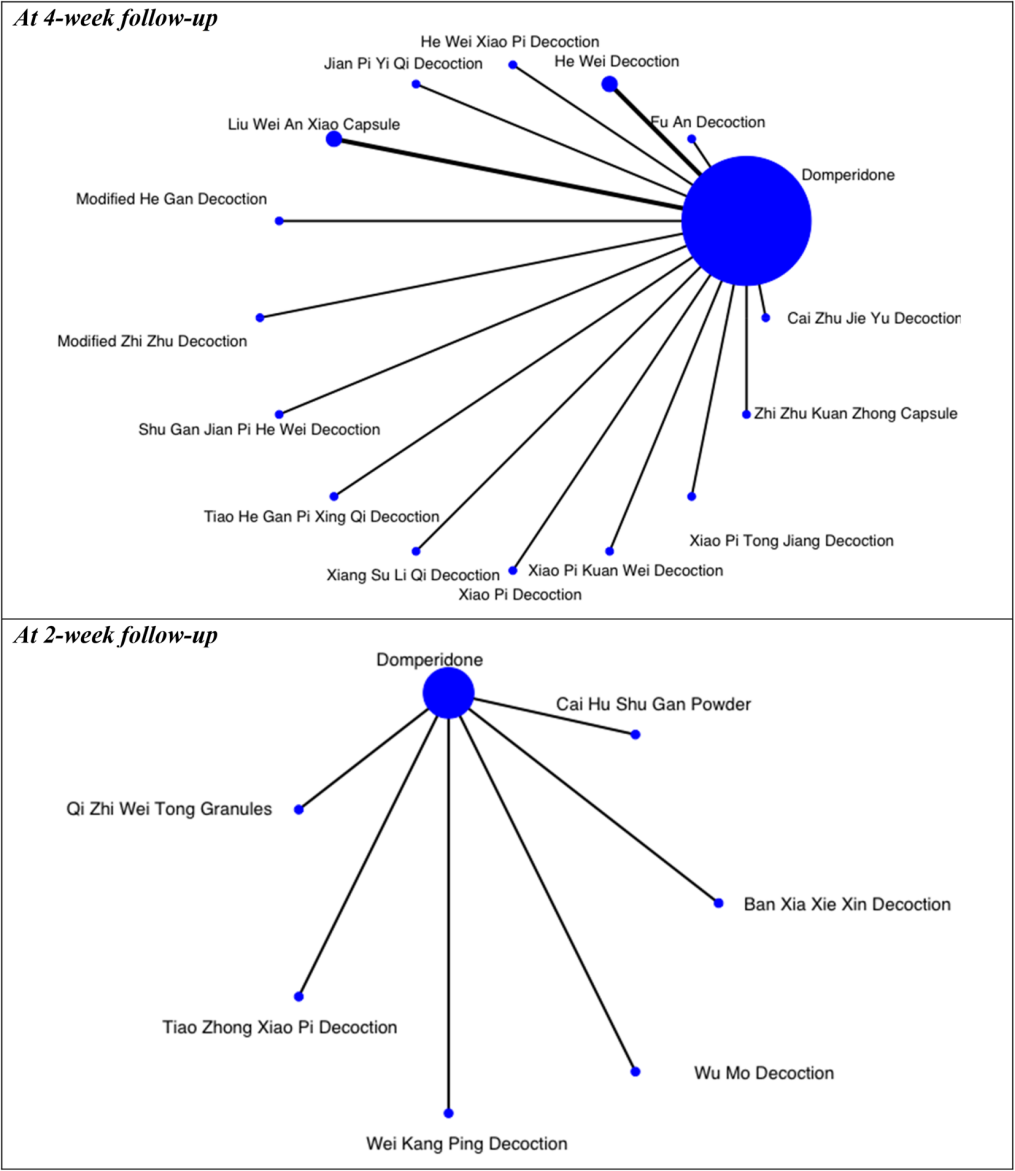
Network of comparisons on alleviation of global symptoms at different follow-up periods: Chinese herbal medicine versus domperidone. The width of the lines represents the proportion of the number of trials for each comparison with the total number of trials, and the size of the nodes represents the proportion of the number of randomised patients (sample sizes)

**Fig. 6:**
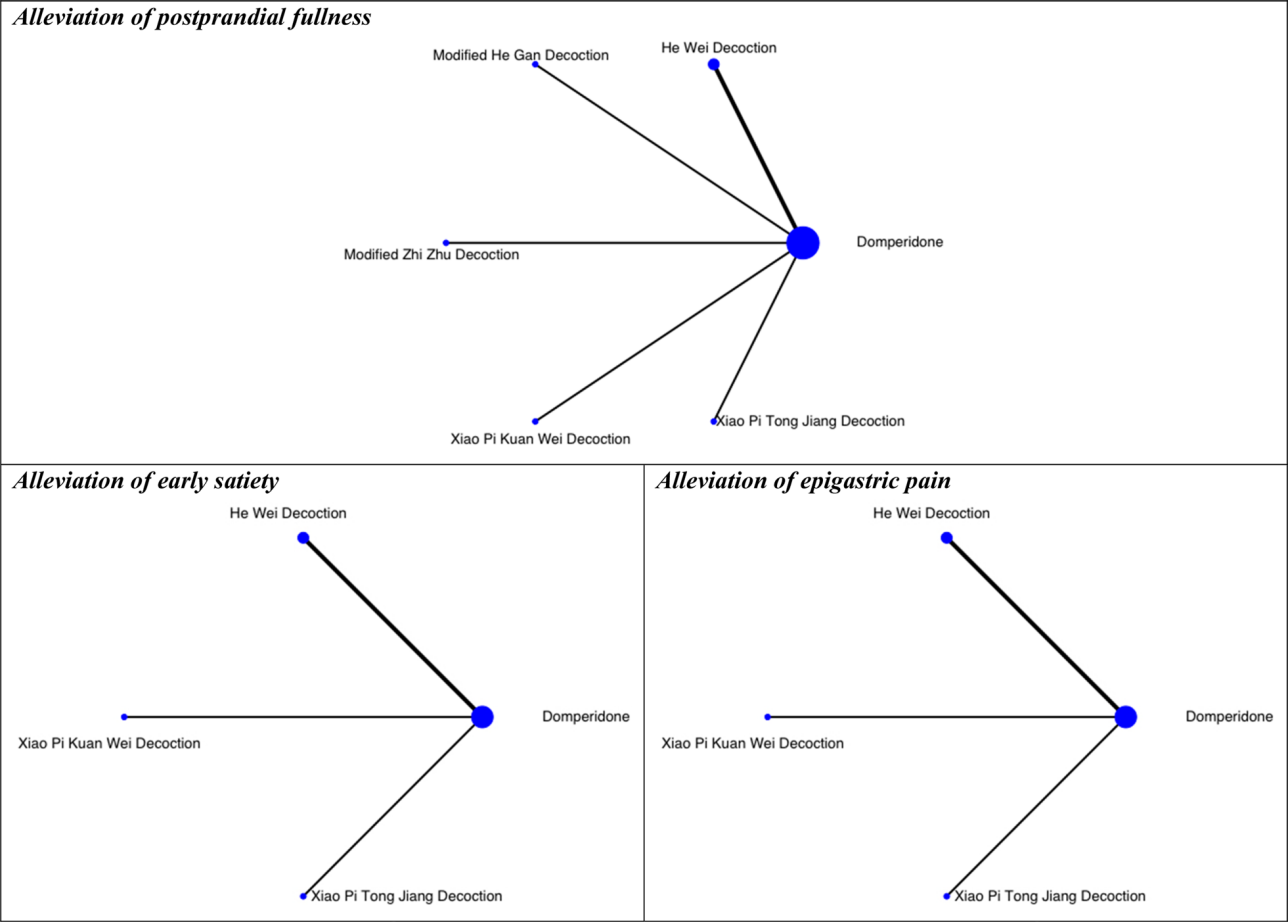
Networks of comparisons on secondary outcomes at 4-week follow-up: Chinese herbal medicine versus domperidone. The width of the lines represents the proportion of the number of trials for each comparison with the total number of trials, and the size of the nodes represents the proportion of the number of randomised patients (sample sizes*)*

**Fig. 7:**
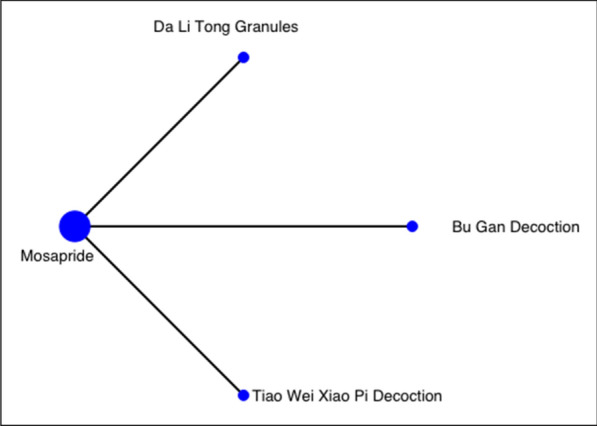
Network of comparisons on alleviation of global symptoms at 4-week follow-up: Chinese herbal medicine versus mosapride. The width of the lines represents the proportion of the number of trials for each comparison with the total number of trials, and the size of the nodes represents the proportion of the number of randomised patients (sample sizes)

Five star-shaped networks were devised to illustrate comparison networks of CHM formulae in alleviating global symptoms, postprandial fullness, early satiety, and epigastric pain against domperidone (Figs. 5, 6, 7). Another star-shaped network was used to illustrate the comparison network of CHM formulae in alleviating global symptoms against mosapride [Fig. 10]. The quality of evidence supporting each network is illustrated in Additional file 1: Appendix 4–9.

#### Alleviation of global symptoms

In the NMA of seventeen RCTs, no specific CHM formula was significantly better than domperidone or other CHM formulae in the network in alleviating global symptoms at 4-week follow-up (Table 5). Nevertheless, according to the partially contextualised framework, Modified Zhi Zhu Decoction (RD: 0.28; 95% CI: − 0.03 to 0.75) may have a moderate beneficial effect in alleviating global symptoms at 4-week, comparing to domperidone. It was the best-ranked intervention in the network (SUCRA: 0.85), as supported by low certainty of evidence (Table 6).

Similarly, in the NMA of six RCTs, no specific CHM formula was significantly superior to domperidone or other CHM formulae in the network in alleviating global symptoms at 2-week. Under the partially contextualised framework, both Cai Hu Shu Gan Powder (RD: 0.16; 95% CI: − 0.06 to 0.50) and Wei Kang Ping Decoction (RD: 0.16; 95% CI: − 0.07 to 0.49) may have a small beneficial effect in alleviating global symptoms at 2-week, as compared to domperidone. They were the best-ranked intervention in the network (SUCRA: 0.79), as supported by low certainty of evidence (Table 7).

**Table 6:** Classification of interventions based on network meta-analysis on alleviating global symptoms at 4-week follow-up: domperidone as comparator

Classification of intervention	CHM formula	Risk difference (95% CI)	Surface under the cumulative ranking curve	Certainty of evidence^+^
Moderate beneficial effect^#^	Modified Zhi Zhu Decoction	0.28 (− 0.03, 0.75)	0.85	Low
Small beneficial effect^†^	Xiao Pi Tong Jiang Decoction	0.15 (− 0.11, 0.57)	0.64	Low
	Xiao Pi Kuan Wei Decoction	0.13 (− 0.11, 0.50)	0.61	Low
	Jian Pi Yi Qi Decoction	0.13 (− 0.11, 0.49)	0.60	Low
	Shu Gan Jian Pi He Wei Decoction	0.12 (− 0.11, 0.49)	0.59	Low
	Xiang Su Li Qi Decoction	0.13 (− 0.11, 0.52)	0.59	Low
	He Wei Decoction	0.10 (− 0.07, 0.34)	0.57	Very low
	Modified He Gan Decoction	0.11 (− 0.12, 0.46)	0.54	Low
	Liu Wei An Xiao Capsule	0.09 (− 0.07, 0.34)	0.53	Very low
	Xiao Pi Decoction	0.09 (− 0.13, 0.44)	0.49	Low
Trivial to no beneficial effect^‡^	He Wei Xiao Pi Decoction	0.07 (− 0.14, 0.38)	0.42	Very low
	Tiao He Gan Pi Xing Qi Decoction	0.06 (− 0.14, 0.38)	0.41	Low
	Fu An Decoction	0.06 (− 0.14, 0.38)	0.40	Low
	Cai Zhu Jie Yu Decoction	0.06 (− 0.15, 0.36)	0.38	Low
	Zhi Zhu Kuan Zhong Capsule	− 0.03 (− 0.19, 0.22)	0.16	Very low

*CHM* Chinese herbal medicine, *CI* Confidence interval

^#^Moderate beneficial effect: 0.31 > risk difference ≥ 0.20

^†^Small beneficial effect: 0.20 > risk difference ≥ 0.08

^‡^Trivial to no beneficial effect: risk difference < 0.08

^+^Quality of evidence ratings for comparisons in network meta-analysis on alleviating global symptoms at 4-week follow-up (domperidone as the comparator). Details refer to Additional file 1: Appendix 4

In the NMA of three RCTs, no specific CHM formula was significantly more effective than mosapride or other CHM formulae in the network in alleviating global symptoms at 2-week (Table 8). According to the partially contextualised framework, Da Li Tong Granules (RD: 0.12; 95% CI: − 0.05 to 0.35) may have a small beneficial effect in alleviating global symptoms at 2-week, when compared to mosapride. It was the best-ranked intervention in the network (SUCRA: 0.85), as supported by low certainty of evidence (Table 9).

**Table 7:** Classification of interventions based on network meta-analysis on alleviating global symptoms at 2-week follow-up: domperidone as the comparator

Classification of intervention	CHM formula	Risk difference (95% CI)	Surface under the cumulative ranking curve	Certainty of evidence^+^
Small beneficial effect^†^	Cai Hu Shu Gan Powder	0.16 (− 0.06, 0.50)	0.79	Low
	Wei Kang Ping Decoction	0.16 (− 0.07, 0.49)	0.79	Low
Trivial to no beneficial effect^‡^	Tiao Zhong Xiao Pi Decoction	0.06 (− 0.11, 0.29)	0.50	Low
	Wu Mo Decoction	0.06 (− 0.12, 0.30)	0.50	Low
	Ban Xia Xie Xin Decoction	0.02 (− 0.15, 0.26)	0.36	Low
	Qi Zhi Wei Tong Granules	0.01 (− 0.14, 0.22)	0.30	Low

*CHM* Chinese herbal medicine, *CI* Confidence interval

^†^Small beneficial effect: 0.20 > risk difference ≥ 0.08

^‡^Trivial to no beneficial effect: risk difference < 0.08

^+^Quality of evidence ratings for comparisons in network meta-analysis on alleviating global symptoms at 2-week follow-up (domperidone as the comparator). Details refer to Additional file 1: Appendix 5

**Table 8:** Comparative effectiveness of different Chinese herbal medicines: mosapride as comparator

Alleviation of global symptoms at 4-week follow-up (shown in RD and 95% CI)						
DLTG						
0.11 (− 0.11, 0.43)	BGD					
0.11 (− 0.10, 0.43)	0.02 (− 0.15, 0.26)	TXD				
0.12 (− 0.05, 0.35)	0.02 (− 0.11, 0.19)	0.01 (− 0.11, 0.17)	MOSA			

*BGD* Bu Gan Decoction, *CI* Confidence interval, *DLTG* Da Li Tong Granules, *MOSA* Mosapride, *RD* Risk difference, *TXD* Tiao Wei Xiao Pi Decoction

#### Alleviation of postprandial fullness

In the NMA of six RCTs, no specific CHM formulae were significantly superior to domperidone or other CHM formulae in alleviating postprandial fullness at 4-week (Table 5). However, based on the partially contextualised framework, Xiao Pi Kuan Wei Decoction (SMD: − 2.14; 95% CrI: − 2.76 to 0.70) may have a large beneficial effect on postprandial fullness alleviation at 4-week follow-up. It was the best-ranked intervention in the network (SUCRA: 0.85), and this finding was supported by low certainty of evidence (Table 10).

**Table 9:** Classification of interventions based on network meta-analysis on alleviating global symptoms at 4-week follow-up: mosapride as comparator

Classification of intervention	CHM formula	Risk difference (95% CI)	Surface under the cumulative ranking curve	Certainty of evidence^+^
Small beneficial effect^†^	Da Li Tong Granules	0.12 (− 0.05, 0.35)	0.85	Low
Trivial to no beneficial effect^‡^	Bu Gan Decoction	0.02 (− 0.11, 0.19)	0.44	Low
	Tiao Wei Xiao Pi Decoction	0.01 (− 0.11, 0.17)	0.39	Low

*CHM* Chinese herbal medicine, *CI* Confidence interval

^†^Small beneficial effect: 0.20 > risk difference ≥ 0.08

^‡^Trivial to no beneficial effect: risk difference < 0.08

^+^Quality of evidence ratings for comparisons in network meta-analysis on alleviating global symptoms at 4-week follow-up (mosapride as the comparator). Details refer to Additional file 1: Appendix 6

**Table 10:** Classification of interventions based on network meta-analyses on secondary outcomes: domperidone as the comparator

Outcome	Classification of intervention	CHM formula	Standardised mean difference (95% CI)	Surface under the cumulative ranking curve	Certainty of evidence^+^
Alleviation of postprandial fullness	Large beneficial effect^@^	Xiao Pi Kuan Wei Decoction	− 2.14 (− 2.76, 0.70)	0.85	Low
	Moderate beneficial effect^#^	Modified Zhi Zhu Decoction	− 0.64 (− 2.18, 0.74)	0.45	Low
	Small beneficial effect^†^	Modified He Gan Decoction	− 0.39 (− 1.55, 0.54)	0.48	Low
		Xiao Pi Tong Jiang Decoction	− 0.34 (− 2.28, 3.30)	0.43	Low
	Trivial to no beneficial effect^‡^	He Wei Decoction	− 0.12 (− 1.68, 0.80)	0.45	Very low
Alleviation of early satiety	Large beneficial effect^@^	Xiao Pi Kuan Wei Decoction	− 3.90 (− 0.68, − 0.42)	0.92	Low
	Moderate beneficial effect^#^	He Wei Decoction	− 0.58 (− 2.69, 2.06)	0.50	Very low
	Trivial to no beneficial effect^‡^	Xiao Pi Tong Jiang Decoction	− 0.04 (− 2.12, 1.46)	0.33	Low
Alleviation of epigastric pain	Large beneficial effect^@^	Xiao Pi Kuan Wei Decoction	− 1.23 (− 1.66, − 0.29)	0.79	Low
	Moderate beneficial effect^#^	He Wei Decoction	− 0.64 (− 1.18, − 0.33)	0.63	Very low
		Xiao Pi Tong Jiang Decoction	− 0.70 (− 1.44, − 0.35)	0.58	Low

*CHM* Chinese herbal medicine, *CI* Confidence interval

^@^Large beneficial effect: standardised mean difference ≤ − 0.80

^#^Moderate beneficial effect: − 0.80 < standardised mean difference ≤ − 0.50

^†^Small beneficial effect: − 0.50 < standardised mean difference ≤ − 0.20

^‡^Trivial to no beneficial effect: standardised mean difference > − 0.20

^+^Quality of evidence ratings for comparisons in network meta-analysis on alleviating postprandial fullness, early satiety, epigastric pain, and epigastric burning, respectively. Details refer to Additional file 1: Appendix 7–9

A negative standardised mean difference indicated an effect favouring Chinese herbal medicine, while a positive standardised mean difference indicated an effect favouring prokinetics

#### Alleviation of early satiety

In the NMA of four RCTs, Xiao Pi Kuan Wei Decoction was significantly more effective than domperidone in alleviating early satiety at 4-week follow-up (SMD: − 3.90; 95% CrI: − 0.68 to − 0.42) (Table 5). Under the partially contextualised framework, Xiao Pi Kuan Wei Decoction may have a large beneficial effect on early satiety alleviation at 4-week follow-up. It was the best-ranked intervention in the network (SUCRA: 0.92), as supported by low certainty of evidence (Table 10).

## Alleviation of epigastric burning

In the NMA of four RCTs, Xiao Pi Kuan Wei Decoction (SMD: − 1.23; 95% CrI: − 1.66 to − 0.29), He Wei Decoction (SMD: − 0.64; 95% CrI: − 1.18 to − 0.33), and Xiao Pi Tong Jiang Decoction (SMD: − 0.70; 95% CrI: − 1.44 to − 0.35) was significantly better than domperidone in alleviating epigastric burning at 4-week (Table 5). According to the partially contextualised framework, Xiao Pi Kuan Wei Decoction may have a large beneficial effect on epigastric burning alleviation at 8-week. It was the best-ranked intervention in the network (SUCRA: 0.79), and the conclusion was supported by low certainty of evidence (Table 10).

### Adverse events

No serious adverse events were reported in all included RCTs (Table 2). An RCT on Liu Wei An Xiao Capsule reported most cases of adverse events (n = 8) [38], which were related to frequent bowel movements. Three cases of mild adverse events, including oral ulcer (n = 2) and diarrhoea (n = 1), were recorded in an RCT on He Wei Decoction [40]. Two cases of mild adverse events, including diarrhoea (n = 1) and dizziness (n = 1), were found in the RCT on Wu Mo Decoction [54]. A case of mild diarrhoea was also reported in the RCT on Fu An Decoction [61].

## Discussion

### Summary of findings

Pairwise meta-analyses showed that CHM was superior to prokinetics in alleviating global symptoms at 2-week and 4-week follow-up, but the magnitude of differences was smaller than relevant MCID values. Although publication bias favouring CHM was detected for the latter outcome, the direction and statistical significance of the result remained unchanged after applying the trim and fill adjustment. Differences exceeding the MCID were observed in other outcomes, with CHM being better than (i) prokinetics in alleviating postprandial fullness and (ii) domperidone alone in alleviating epigastric pain, at 4-week follow-up. These imply that CHM may well serve as an alternative to prokinetics, given the fact that its effectiveness is similar, if not more effective, to the conventional therapy, especially in alleviating postprandial fullness and epigastric pain. Indeed, they are the main symptoms of PDS and EPS respectively.

As interpreted under the partially contextualised framework, NMAs illustrated that Modified Zhi Zhu Decoction may have a moderate beneficial effect on alleviating global symptoms at 4-week follow-up, while Xiao Pi Kuan Wei Decoction may have a large beneficial effect on alleviating postprandial fullness, early satiety, and epigastric pain. In future guideline revisions, Modified Zhi Zhu Decoction and Xiao Pi Kuan Wei Decoction may be recommended as alternative options for FD patients who are unresponsive to prokinetics or opt-out of the treatment due to associated adverse effects. Additional considerations on the implementation will involve the key aspects below.

### Implications for practice

#### Positioning of Chinese herbal medicine in functional dyspepsia clinical guidelines

Current Asian clinical guideline [8] recommends prokinetics as the first-line treatment for the FD diagnostic subtype of PDS and the second-line treatment after PPIs for EPS. Unfortunately, prokinetics have a relatively high number needed to treat of seven to twelve, and evidence supporting their effectiveness was of very low-quality evidence [11]. Specific prokinetics are also related to serious adverse events. For instance, domperidone and cisapride may trigger life-threatening arrhythmia in patients with cardiovascular conditions [4, 12], and metoclopramide may induce dystonia, parkinsonism-type movements, and tardive dyskinesia [4].

Results of this review suggest that CHM may be a potential substitution to prokinetics. Beyond effectiveness and safety, guideline developers should also consider other criteria in the GRADE Evidence to Decision (EtD) framework [65], including acceptability, feasibility, outcome importance, cost-effectiveness, and equity, when preparing guideline updates. Issues on acceptability and feasibility will be discussed briefly.

#### Acceptability of Chinese herbal medicine among patients with gastrointestinal disorders

CHM is one of the most utilised modalities of traditional, complementary, and integrative medicine (TCIM) worldwide, particularly in Asia [14]. In Taiwan, more than 60% of the population utilised TCM services on a regular basis [66]. 86% of those services involved CHM prescriptions [66]. In Singapore, over three-quarters of the population used TCIM at least once a year, and CHM was the most popular TCIM modality [67]. In both healthcare systems, patients with gastrointestinal disorders constituted a significant portion of CHM users [66, 67]. With a high prevalence of both CHM utilisation and FD in Asia, the acceptability of CHM for FD treatment is likely to be high. The use of CHM may also be accepted by patients in Canada [68] and Australia [69], where TCM practice is statutorily regulated.

#### Feasibility of Chinese herbal medicine utilisation in interprofessional environment

If CHM is to be included in the next FD clinical guideline, interprofessional collaboration between conventional clinicians and TCM clinicians will be required for the implementation: a conventional clinician may refer a patient unresponsive to prokinetics to a TCM clinician, and vice versa when CHM is found to be ineffective. Different referral mechanisms have been devised to clarify the respective duties and responsibilities of conventional clinicians and TCM clinicians, of which suitability would depend on the clinical context [70]. Potential malpractice liability related to adverse events is a key barrier for collaboration [71]. To address this barrier, pharmacovigilance mechanisms for monitoring potential CHM-related adverse events should be in place to improve confidence in interprofessional collaboration.

### Implications for research

Based on the best available evidence, Modified Zhi Zhu Decoction and Xiao Pi Kuan Wei Decoction may be the best CHM formulae for alleviating FD global and individual symptoms (postprandial fullness, early satiety, epigastric pain, and epigastric burning), respectively. Modern pharmacology may help explain their therapeutic effects. In Modified Zhi Zhu Decoction [48], the main ingredients are Aurantii Fructus Immaturus (Zhishi) and Rhizoma Atractylodis Macrocephala (Baizhu). Both herbs contain two flavonoids, naringin and hesperidin, which can be converted to naringenin and hesperitin in human body and may alleviate dyspeptic symptoms through increasing gastrointestinal motility [72, 73]. In Xiao Pi Kuan Wei Decoction [43], the main ingredient of Radix Bupleuri (Chaihu) contains saikosaponin, a component with antidepressant-like effects [74]. It may relieve dyspeptic symptoms via addressing disorder at the brain-gut axis [75]. Aurantii Fructus Immaturus and Rhizoma Atractylodis Macrocephala in the decoction may also help enhance therapeutic effects by increasing the motility of gastrointestinal tract.

In the future, confirmatory head-to-head RCTs should be carried out to further investigate their comparative effectiveness against prokinetics. Trialists should beware of several design aspects when planning such trials:

#### Patient eligibility

Validated Rome IV diagnostic criteria for FD [1] should be adopted as the eligibility criteria to enable comparisons between and synthesis of similar trials. Patients who remain to be symptomatic after receiving PPIs should be recruited as they are refractory to the current first-line therapy of the North American guideline [4].

#### Interventions and comparisons

Three-arm RCTs consisting of Modified Zhi Zhu Decoction, Xiao Pi Kuan Wei Decoction, and prokinetics should be performed to allow head-to-head comparisons between the two formulae, and against prokinetics. The choice of prokinetics should comply with local regulatory requirements and safety profile.

#### Outcome measures

An array of expert-recommended endpoints for FD trials should be adopted to capture outcome changes in a multifaceted manner. Global symptom alleviation evaluated on dichotomous scale allows a global assessment [21], which can be supplemented with assessment on individual symptoms on a seven-point Likert scale [76]. Nepean Dyspepsia Index [77] provides information on changes in disease-specific quality of life [21, 76]. Objective measurements, including gastric emptying scintigraphy, gastric barostat study, and liquid nutrient drink test, may also be conducted to supplement patient-reported outcomes [21, 76]. Furthermore, a longer follow-up period of forty-eight weeks is recommended to allow sufficient assessment on refractory FD, in which symptoms usually wax and wane [76].

To minimise selection bias, trialists should allocate interventions to participants based on random number table or generator and conceal the allocation sequence from research personnel [26]. Blinding of participants and research personnel also helps reduce performance bias and detection bias of RCTs [26]. A priori protocols should be published and followed to avoid outcome reporting bias [26]. Furthermore, to improve the transparency of RCTs, trialists should report their studies in accordance with the CONSORT (Consolidated Standards of Reporting Trials) Extension for CHM Formulas [78].

In this study, specific TCM diagnostic patterns were used in fifteen out of twenty-eight included RCTs as part of the inclusion criteria, despite the absence of gold standards for pattern differentiation [79, 80]. Having said that, when evidence-based differentiation rules are established in the future, clinical trials can be carried out to compare the effectiveness of different CHM formulae that are targeting the same TCM diagnostic patterns [81].

## Limitations

This study has several limitations. First, due to the small number of included trials, funnel plots or relevant statistical tests were not conducted for all outcomes to examine the possible presence of publication bias. Second, the consistency of NMA results could not be assessed since no comparisons between CHM formulae were supported by both direct (head-to-head comparisons between CHM formulae) and indirect evidence (comparisons between CHM formulae and prokinetics). Third, sample size of the included RCTs was small and might have influenced the precision of our results. We assessed the degree of imprecision for each outcome under the GRADE framework (Tables 3, 4). The potential impact originating from imprecision has been reflected by downgrading the quality of evidence and certainty of evidence in the GRADE evidence rating for pairwise meta-analyses and NMAs, respectively. Clinicians and policy-makers should consider this information in the decision-making process.

## Conclusions

Results from this review suggested that CHM could be a potential alternative to prokinetics as a first-line treatment for FD or a second-line treatment after PPI. Repositioning of CHM in clinical guidelines require thorough discussions on aspects stipulated in the GRADE EtD framework. Confirmatory head-to-head trials are necessary for evaluating the comparative effectiveness of prokinetics, Modified Zhi Zhu Decoction, and Xiao Pi Kuan Wei Decoction.

## Supplementary Information

**Additional file 1: Appendix 1**. Search strategies for databases. **Appendix 2**. Risk of bias of included studies. **Appendix 3**. (a) Contour-enhanced funnel plot of 20 included studies (all prokinetics). (b) Contour-enhanced funnel plot of 17 included studies (domperidone only). **Appendix 4**. Effect estimates and quality of evidence ratings for comparisons in network meta-analysis on alleviating global symptoms at 4-week follow-up: domperidone as the comparator. **Appendix 5**. Effect estimates and quality of evidence ratings for comparisons in network meta-analysis on alleviating global symptoms at 2-week follow-up: domperidone as the comparator. **Appendix 6**. Effect estimates and quality of evidence ratings for comparisons in network meta-analysis on alleviating global symptoms at 4-week follow-up: mosapride as the comparator. **Appendix 7**. Effect estimates and quality of evidence ratings for comparisons in network meta-analysis on alleviating postprandial fullness: domperidone as the comparator. **Appendix 8**. Effect estimates and quality of evidence ratings for comparisons in network meta-analysis on alleviating early satiety: domperidone as the comparator. **Appendix 9**. Effect estimates and quality of evidence ratings for comparisons in network meta-analysis on alleviating epigastric pain: domperidone as the comparator.

## Data Availability

The datasets used and/or analysed during the current study are available from the corresponding author on reasonable request.

## Abbreviations

CHM: Chinese herbal medicine
CI: Confidence interval
Crl: Credible interval
EPS: Epigastric pain syndrome
EtD: Evidence to Decision
FD: Functional dyspepsia
GRADE: Grading of Recommendations Assessment, Development and Evaluation
MCID: Minimally clinically important difference
NMA: Network meta-analysis
PDS: Postprandial distress syndrome
PPI: Proton pump inhibitor
RCT: Randomised controlled trials
RD: Risk difference
RR: Risk ratio
SMD: Standardised mean difference
SUCRA: Surface under the cumulative ranking curve
TCIM: Traditional, complementary, and integrative medicine
TCM: Traditional Chinese Medicine
