# Supplementary material for: Chinese herbal medicine for functional dyspepsia: a network meta-analysis of prokinetic-controlled randomised trials
Source: Chin Med. 2021 Dec 20;16:140. doi: 10.1186/s13020-021-00556-6 (PMC8691044; doi:10.1186/s13020-021-00556-6)
Supplement: Supplementary file 1 — Additional file 1: Appendix 1. Search strategies for databases. Appendix 2. Risk of bias of included studies. Appendix 3. (a) Contour-enhanced funnel plot of 20 included studies (all prokinetics). (b) Contour-enhanced funnel plot of 17 included studies (domperidone only). Appendix 4. Effect estimates and quality of evidence ratings for comparisons in network meta-analysis on alleviating global symptoms at 4-week follow-up: domperidone as the comparator. Appendix 5. Effect estimates and quality of evidence ratings for comparisons in network meta-analysis on alleviating global symptoms at 2-week follow-up: domperidone as the comparator. Appendix 6. Effect estimates and quality of evidence ratings for comparisons in network meta-analysis on alleviating global symptoms at 4-week follow-up: mosapride as the comparator. Appendix 7. Effect estimates and quality of evidence ratings for comparisons in network meta-analysis on alleviating postprandial fullness: domperidone as the comparator. Appendix 8. Effect estimates and quality of evidence ratings for comparisons in network meta-analysis on alleviating early satiety: domperidone as the comparator. Appendix 9. Effect estimates and quality of evidence ratings for comparisons in network meta-analysis on alleviating epigastric pain: domperidone as the comparator. [file 13020_2021_556_MOESM1_ESM.docx]

**Appendix 1.** Search strategies for databases

1. Wanfang Data from inception to 8 July 2020:

关键词:("中药" OR "中草药" OR "中成药" OR "中西医" OR "中医") AND 关键词:("功能性消化不良" OR "消化不良") AND 摘要:("随机") yielded 767 citations.

1. China National Knowledge Infrastructure from inception to 8 July 2020:

(KY='中药' OR KY='中草药' OR KY='中成药' OR KY='中西医' OR KY='中医') AND (KY='功能性消化不良' OR KY='消化不良') AND (AB='随机') yielded 180 citations.

1. SinoMed from inception to 8 July 2020:

("功能性消化不良"[关键词] OR "消化不良"[关键词]) AND ("中药"[关键词] OR "中草药"[关键词] OR "中成药"[关键词] OR "中西医"[关键词] OR "中医"[关键词]) AND ("随机"[摘要]) yielded 78 citations.

1. Index to Taiwan Periodical Literature System from inception to 8 July 2020:

(TX= 中藥 OR 中草藥 OR 中成藥 OR 中西醫 OR 中醫) [AND] (TX= 功能性消化不良 OR 消化不良) [AND] (TX= 隨機) yielded 12 citations.

1. MEDLINE from inception to 8 July 2020:

| **#** | **Search Statement** | **Results** |
| --- | --- | --- |
| 1 | clinical trial.mp. | 684720 |
| 2 | clinical trial.pt. | 523145 |
| 3 | random:.mp. | 1195453 |
| 4 | 1 or 2 or 3 | 1567968 |
| 5 | exp Dyspepsia/ | 8595 |
| 6 | dyspepsia*.mp. | 12563 |
| 7 | functional dyspepsia*.mp. | 2654 |
| 8 | non ulcer dyspepsia*.mp. | 1010 |
| 9 | 5 or 6 or 7 or 8 | 12563 |
| 10 | exp Drugs, Chinese Herbal/ | 43296 |
| 11 | Chinese herb*.mp. | 46586 |
| 12 | exp Medicine, Chinese Traditional/ | 18984 |
| 13 | Traditional Chinese medic*.mp. | 18154 |
| 14 | exp Phytotherapy/ | 39198 |
| 15 | phytother*.mp. | 39326 |
| 16 | (chinese adj5 (traditional or medic*)).mp. | 42197 |
| 17 | (herbs or herbal).mp. | 70700 |
| 18 | (plant or plants).mp. | 718241 |
| 19 | (traditional adj5 medic*).mp. | 59952 |
| 20 | 10 or 11 or 12 or 13 or 14 or 15 or 16 or 17 or 18 or 19 | 800018 |
| 21 | 4 and 9 and 20 | 179 |
| 22 | limit 21 to humans | 167 |

1. EMBASE from inception to 8 July 2020:

| **#** | **Search Statement** | **Results** |
| --- | --- | --- |
| 1 | random:.tw. | 1547901 |
| 2 | clinical trial:.mp. | 1714776 |
| 3 | exp health care quality/ | 3202723 |
| 4 | 1 or 2 or 3 | 5290966 |
| 5 | exp dyspepsia/ | 33717 |
| 6 | dyspepsia*.mp. | 36663 |
| 7 | functional dyspepsia*.mp. | 5574 |
| 8 | non ulcer dyspepsia*.mp. | 1404 |
| 9 | 5 or 6 or 7 or 8 | 36663 |
| 10 | exp Chinese medicine/ | 50648 |
| 11 | exp oriental medicine/ | 2617 |
| 12 | exp herbaceous agent/ | 51346 |
| 13 | exp medicinal plant/ | 237892 |
| 14 | exp Chinese herb/ | 4813 |
| 15 | Chinese medic*.mp. | 65752 |
| 16 | oriental medic*.mp. | 3968 |
| 17 | herbaceous agent.mp. | 51348 |
| 18 | medicinal plant*.mp. | 95741 |
| 19 | Chinese herb*.mp. | 16602 |
| 20 | herb*.mp. | 187082 |
| 21 | 10 or 11 or 12 or 13 or 14 or 15 or 16 or 17 or 18 or 19 or 20 | 423725 |
| 22 | 4 and 9 and 21 | 720 |
| 23 | limit 22 to humans | 676 |

1. Cochrane Central Register of Controlled Trials from inception to 8 July 2020:

| **#** | **Search Statement** | **Results** |
| --- | --- | --- |
| 1 | MeSH descriptor: [Dyspepsia] explode all trees | 1097 |
| 2 | MeSH descriptor: [Medicine, Chinese Traditional] explode all trees | 1137 |
| 3 | MeSH descriptor: [Herbal Medicine] explode all trees | 59 |
| 4 | MeSH descriptor: [Drugs, Chinese Herbal] explode all trees | 3516 |
| 5 | MeSH descriptor: [Plants, Medicinal] explode all trees | 936 |
| 6 | MeSH descriptor: [Phytotherapy] explode all trees | 4137 |
| 7 | MeSH descriptor: [Medicine, East Asian Traditional] explode all trees | 1250 |
| 8 | #2 or #3 or #4 or #5 or #6 or #7 | 7420 |
| 9 | #1 and #8, in Trials | 47 |

**Appendix 2.** Risk of bias of included studies

| **Reference** | **Domains** | | | | | **Overall** |
| --- | --- | --- | --- | --- | --- | --- |
|  | Randomisation process | Deviations from intended interventions | Missing outcome data | Measurement of the outcome | Selection of the reported result |  |
| Gong, 2012 | **High risk:** Details on allocation sequence generation and baseline differences between intervention groups were not reported. | **Some concerns:** Blinding was not available for participants, carers, and people delivering the interventions. | **Low risk:** Outcome data were available for nearly all participants randomised. | **Low risk:** Methods of measuring the outcomes were appropriate. Measurement of the outcomes could not have differed between intervention groups. | **Some concerns:** Details on whether the data were analysed following a pre-specified analysis plan were not reported. | **High risk** |
| Li, 2004 | **High risk:** Details on allocation sequence generation and baseline differences between intervention groups were not reported. | **Some concerns:** Blinding was not available for participants, carers, and people delivering the interventions. | **Low risk:** Outcome data were available for nearly all participants randomised. | **Low risk:** Methods of measuring the outcomes were appropriate. Measurement of the outcomes could not have differed between intervention groups. | **Some concerns:** Details on whether the data were analysed following a pre-specified analysis plan were not reported. | **High risk** |
| Zhou, 2016 | **Some concerns:** Details on allocation sequence generation were not reported. | **Some concerns:** Blinding was not available for participants, carers, and people delivering the interventions. | **Low risk:** Outcome data were available for nearly all participants randomised. | **Low risk:** Methods of measuring the outcomes were appropriate. Measurement of the outcomes could not have differed between intervention groups. | **Some concerns:** Details on whether the data were analysed following a pre-specified analysis plan were not reported. | **Some concerns** |
| Leng, 2014 | **Some concerns:** Details on allocation sequence generation were not reported. | **Some concerns:** Blinding was not available for participants, carers, and people delivering the interventions. | **Low risk:** Outcome data were available for nearly all participants randomised. | **Low risk:** Methods of measuring the outcomes were appropriate. Measurement of the outcomes could not have differed between intervention groups. | **Some concerns:** Details on whether the data were analysed following a pre-specified analysis plan were not reported. | **Some concerns** |
| Gao, 2013 | **Some concerns:** Details on allocation sequence generation were not reported. | **Some concerns:** Blinding was not available for participants, carers, and people delivering the interventions. | **Low risk:** Outcome data were available for nearly all participants randomised. | **Low risk:** Methods of measuring the outcomes were appropriate. Measurement of the outcomes could not have differed between intervention groups. | **Some concerns:** Details on whether the data were analysed following a pre-specified analysis plan were not reported. | **Some concerns** |
| Liu, 2010 | **Some concerns:** Details on allocation sequence generation were not reported. | **Some concerns:** Blinding was not available for participants, carers, and people delivering the interventions. | **Low risk:** Outcome data were available for nearly all participants randomised. | **Low risk:** Methods of measuring the outcomes were appropriate. Measurement of the outcomes could not have differed between intervention groups. | **Some concerns:** Details on whether the data were analysed following a pre-specified analysis plan were not reported. | **Some concerns** |
| Lai, 2019 | **Some concerns:** Details on allocation sequence generation were not reported. | **Some concerns:** Blinding was not available for participants, carers, and people delivering the interventions. | **Low risk:** Outcome data were available for nearly all participants randomised. | **Low risk:** Methods of measuring the outcomes were appropriate. Measurement of the outcomes could not have differed between intervention groups. | **Some concerns:** Details on whether the data were analysed following a pre-specified analysis plan were not reported. | **Some concerns** |
| Cai, 2019 | **Some concerns:** Details on allocation sequence generation were not reported. | **Some concerns:** Blinding was not available for participants, carers, and people delivering the interventions. | **Low risk:** Outcome data were available for nearly all participants randomised. | **Low risk:** Methods of measuring the outcomes were appropriate. Measurement of the outcomes could not have differed between intervention groups. | **Some concerns:** Details on whether the data were analysed following a pre-specified analysis plan were not reported. | **Some concerns** |
| Dong, 2011 | **Some concerns:** Details on allocation sequence generation were not reported. | **Some concerns:** Blinding was not available for participants, carers, and people delivering the interventions. | **Low risk:** Outcome data were available for nearly all participants randomised. | **Low risk:** Methods of measuring the outcomes were appropriate. Measurement of the outcomes could not have differed between intervention groups. | **Some concerns:** Details on whether the data were analysed following a pre-specified analysis plan were not reported. | **Some concerns** |
| Liu, 2012 | **High risk:** Details on allocation sequence generation and baseline differences between intervention groups were not reported. | **Some concerns:** Blinding was not available for participants, carers, and people delivering the interventions. | **Low risk:** Outcome data were available for nearly all participants randomised. | **Low risk:** Methods of measuring the outcomes were appropriate. Measurement of the outcomes could not have differed between intervention groups. | **Some concerns:** Details on whether the data were analysed following a pre-specified analysis plan were not reported. | **High risk** |
| Liu, 2015 | **Some concerns:** Details on allocation sequence generation were not reported. | **Some concerns:** Blinding was not available for participants, carers, and people delivering the interventions. | **Low risk:** Outcome data were available for nearly all participants randomised. | **Low risk:** Methods of measuring the outcomes were appropriate. Measurement of the outcomes could not have differed between intervention groups. | **Some concerns:** Details on whether the data were analysed following a pre-specified analysis plan were not reported. | **Some concerns** |
| Ma, 2014 | **High risk:** Details on allocation sequence generation and baseline differences between intervention groups were not reported. | **Some concerns:** Blinding was not available for participants, carers, and people delivering the interventions. | **Low risk:** Outcome data were available for nearly all participants randomised. | **Low risk:** Methods of measuring the outcomes were appropriate. Measurement of the outcomes could not have differed between intervention groups. | **Some concerns:** Details on whether the data were analysed following a pre-specified analysis plan were not reported. | **High risk** |
| Duan, 2013 | **Some concerns:** Details on allocation sequence generation were not reported. | **Some concerns:** Blinding was not available for participants, carers, and people delivering the interventions. | **Low risk:** Outcome data were available for nearly all participants randomised. | **Low risk:** Methods of measuring the outcomes were appropriate. Measurement of the outcomes could not have differed between intervention groups. | **Some concerns:** Details on whether the data were analysed following a pre-specified analysis plan were not reported. | **Some concerns** |
| Wang, 2012 | **Some concerns:** Details on allocation sequence generation were not reported. | **Some concerns:** Blinding was not available for participants, carers, and people delivering the interventions. | **Low risk:** Outcome data were available for nearly all participants randomised. | **Low risk:** Methods of measuring the outcomes were appropriate. Measurement of the outcomes could not have differed between intervention groups. | **Some concerns:** Details on whether the data were analysed following a pre-specified analysis plan were not reported. | **Some concerns** |
| Li, 2014 | **Some concerns:** Details on allocation sequence generation were not reported. | **Some concerns:** Blinding was not available for participants, carers, and people delivering the interventions. | **Low risk:** Outcome data were available for nearly all participants randomised. | **Low risk:** Methods of measuring the outcomes were appropriate. Measurement of the outcomes could not have differed between intervention groups. | **Some concerns:** Details on whether the data were analysed following a pre-specified analysis plan were not reported. | **Some concerns** |
| Huang, 2017 | **Some concerns:** Details on allocation sequence generation were not reported. | **Some concerns:** Blinding was not available for participants, carers, and people delivering the interventions. | **Low risk:** Outcome data were available for nearly all participants randomised. | **Low risk:** Methods of measuring the outcomes were appropriate. Measurement of the outcomes could not have differed between intervention groups. | **Some concerns:** Details on whether the data were analysed following a pre-specified analysis plan were not reported. | **Some concerns** |
| Sheng, 2016 | **Some concerns:** Details on allocation sequence generation were not reported. | **Some concerns:** Blinding was not available for participants, carers, and people delivering the interventions. | **Low risk:** Outcome data were available for nearly all participants randomised. | **Low risk:** Methods of measuring the outcomes were appropriate. Measurement of the outcomes could not have differed between intervention groups. | **Some concerns:** Details on whether the data were analysed following a pre-specified analysis plan were not reported. | **Some concerns** |
| Zhao, 2013 | **Some concerns:** Details on allocation sequence generation were not reported. | **Some concerns:** Blinding was not available for participants, carers, and people delivering the interventions. | **Low risk:** Outcome data were available for nearly all participants randomised. | **Low risk:** Methods of measuring the outcomes were appropriate. Measurement of the outcomes could not have differed between intervention groups. | **Some concerns:** Details on whether the data were analysed following a pre-specified analysis plan were not reported. | **Some concerns** |
| Liu, 2016 | **Some concerns:** Details on allocation sequence generation were not reported. | **Some concerns:** Blinding was not available for participants, carers, and people delivering the interventions. | **Low risk:** Outcome data were available for nearly all participants randomised. | **Low risk:** Methods of measuring the outcomes were appropriate. Measurement of the outcomes could not have differed between intervention groups. | **Some concerns:** Details on whether the data were analysed following a pre-specified analysis plan were not reported. | **Some concerns** |
| Ma, 2013 | **Some concerns:** Details on allocation sequence generation were not reported. | **Some concerns:** Blinding was not available for participants, carers, and people delivering the interventions. | **Low risk:** Outcome data were available for nearly all participants randomised. | **Low risk:** Methods of measuring the outcomes were appropriate. Measurement of the outcomes could not have differed between intervention groups. | **Some concerns:** Details on whether the data were analysed following a pre-specified analysis plan were not reported. |  |
| Liu, 2008 | **Some concerns:** Details on allocation sequence generation were not reported. | **Some concerns:** Blinding was not available for participants, carers, and people delivering the interventions. | **Low risk:** Outcome data were available for nearly all participants randomised. | **Low risk:** Methods of measuring the outcomes were appropriate. Measurement of the outcomes could not have differed between intervention groups. | **Some concerns:** Details on whether the data were analysed following a pre-specified analysis plan were not reported. | **Some concerns** |
| Wang, 2006 | **Some concerns:** Details on allocation sequence generation were not reported. | **Some concerns:** Blinding was not available for participants, carers, and people delivering the interventions. | **Low risk:** Outcome data were available for nearly all participants randomised. | **Low risk:** Methods of measuring the outcomes were appropriate. Measurement of the outcomes could not have differed between intervention groups. | **Some concerns:** Details on whether the data were analysed following a pre-specified analysis plan were not reported. | **Some concerns** |
| Hu, 2006 | **Some concerns:** Details on allocation sequence generation were not reported. | **Some concerns:** Blinding was not available for participants, carers, and people delivering the interventions. | **Low risk:** Outcome data were available for nearly all participants randomised. | **Low risk:** Methods of measuring the outcomes were appropriate. Measurement of the outcomes could not have differed between intervention groups. | **Some concerns:** Details on whether the data were analysed following a pre-specified analysis plan were not reported. | **Some concerns** |
| Chen, 2016 | **Some concerns:** Details on allocation sequence generation were not reported. | **Some concerns:** Blinding was not available for participants, carers, and people delivering the interventions. | **Low risk:** Outcome data were available for nearly all participants randomised. | **Low risk:** Methods of measuring the outcomes were appropriate. Measurement of the outcomes could not have differed between intervention groups. | **Some concerns:** Details on whether the data were analysed following a pre-specified analysis plan were not reported. | **Some concerns** |
| Wang, 2015 | **Some concerns:** Details on allocation sequence generation were not reported. | **Some concerns:** Blinding was not available for participants, carers, and people delivering the interventions. | **Low risk:** Outcome data were available for nearly all participants randomised. | **Low risk:** Methods of measuring the outcomes were appropriate. Measurement of the outcomes could not have differed between intervention groups. | **Some concerns:** Details on whether the data were analysed following a pre-specified analysis plan were not reported. | **Some concerns** |
| Huang, 2010 | **Some concerns:** Details on allocation sequence generation were not reported. | **Some concerns:** Blinding was not available for participants, carers, and people delivering the interventions. | **Low risk:** Outcome data were available for nearly all participants randomised. | **Low risk:** Methods of measuring the outcomes were appropriate. Measurement of the outcomes could not have differed between intervention groups. | **Some concerns:** Details on whether the data were analysed following a pre-specified analysis plan were not reported. | **Some concerns** |
| Zheng, 2010 | **Some concerns:** Details on allocation sequence generation were not reported. | **Some concerns:** Blinding was not available for participants, carers, and people delivering the interventions. | **Low risk:** Outcome data were available for nearly all participants randomised. | **Low risk:** Methods of measuring the outcomes were appropriate. Measurement of the outcomes could not have differed between intervention groups. | **Some concerns:** Details on whether the data were analysed following a pre-specified analysis plan were not reported. | **Some concerns** |
| Liu, 2013 | **Some concerns:** Details on allocation sequence generation were not reported. | **Some concerns:** Blinding was not available for participants, carers, and people delivering the interventions. | **Low risk:** Outcome data were available for nearly all participants randomised. | **Low risk:** Methods of measuring the outcomes were appropriate. Measurement of the outcomes could not have differed between intervention groups. | **Some concerns:** Details on whether the data were analysed following a pre-specified analysis plan were not reported. | **Some concerns** |

**Appendix 3a.** Contour-enhanced funnel plot of 20 included studies (all prokinetics)

| **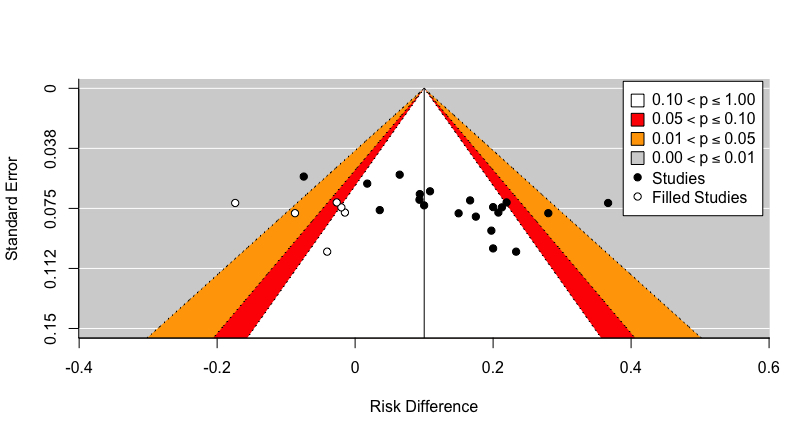** |
| --- |

*X-axis corresponds to risk difference for the effect of Chinese herbal medicine in alleviating global symptoms at 4-week follow-up, as compared to all prokinetics. Y-axis represents the standard error of risk difference. A positive risk difference indicated an effect favouring Chinese herbal medicine, while a negative risk difference indicated an effect favouring prokinetics. As there is a suggestion of missing studies on the left-hand side of the plot, publication bias favouring Chinese herbal medicine may exist. Trim and fill adjustment for publication bias is applied.*

**Appendix 3b.** Contour-enhanced funnel plot of 17 included studies (domperidone only)

| 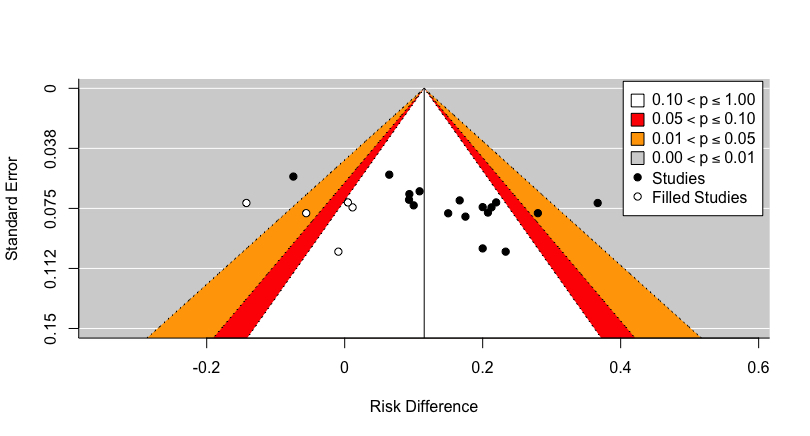 |
| --- |

*X-axis corresponds to risk difference for the effect of Chinese herbal medicine in alleviating global symptoms at 4-week follow-up, as compared to domperidone. Y-axis represents the standard error of risk difference. A positive risk difference indicated an effect favouring Chinese herbal medicine, while a negative risk difference indicated an effect favouring domperidone. As there is a suggestion of missing studies on the left-hand side of the plot, publication bias favouring Chinese herbal medicine may exist. Trim and fill adjustment for publication bias is applied.*

**Appendix 4.** Effect estimates and quality of evidence ratings for comparisons in network meta-analysis on alleviating global symptoms at 4-week follow-up: domperidone as the comparator

| **Comparison** | **Direct evidence** | | **Indirect evidence** | | **Network meta-analysis** | |
| --- | --- | --- | --- | --- | --- | --- |
|  | Risk ratio (95% confidence interval) | Quality of evidence | Risk ratio (95% credible interval) | Quality of evidence^@^ | Risk ratio (95% credible interval) | Quality of evidence^#^ |
| Modified Zhi Zhu Decoction *v* Zhi Zhu Kuan Zhong Capsule | — | — | 1.91 (0.85, 3.77) | ⨁◯◯◯  Very low | 1.91 (0.85, 3.77) | ⨁◯◯◯  Very low |
| Xiao Pi Tong Jiang Decoction *v* Zhi Zhu Kuan Zhong Capsule | — | — | 1.57 (0.69, 3.14) | ⨁◯◯◯  Very low | 1.57 (0.69, 3.14) | ⨁◯◯◯  Very low |
| Xiao Pi Kuan Wei Decoction *v* Zhi Zhu Kuan Zhong Capsule | — | — | 1.51 (0.68, 2.94) | ⨁◯◯◯  Very low | 1.51 (0.68, 2.94) | ⨁◯◯◯  Very low |
| Jian Pi Yi Qi Decoction *v* Zhi Zhu Kuan Zhong Capsule | — | — | 1.50 (0.68, 2.94) | ⨁◯◯◯  Very low | 1.50 (0.68, 2.94) | ⨁◯◯◯  Very low |
| Shu Gan Jian Pi He Wei Decoction *v* Zhi Zhu Kuan Zhong Capsule | — | — | 1.49 (0.67, 2.91) | ⨁◯◯◯  Very low | 1.49 (0.67, 2.91) | ⨁◯◯◯  Very low |
| Xiang Su Li Qi Decoction *v* Zhi Zhu Kuan Zhong Capsule | — | — | 1.50 (0.66, 3.00) | ⨁◯◯◯  Very low | 1.50 (0.66, 3.00) | ⨁◯◯◯  Very low |
| He Wei Decoction *v* Zhi Zhu Kuan Zhong Capsule | — | — | 1.42 (0.73, 2.57) | ⨁◯◯◯  Very low | 1.42 (0.73, 2.57) | ⨁◯◯◯  Very low |
| Modified He Gan Decoction *v* Zhi Zhu Kuan Zhong Capsule | — | — | 1.43 (0.64, 2.82) | ⨁◯◯◯  Very low | 1.43 (0.64, 2.82) | ⨁◯◯◯  Very low |
| Liu Wei An Xiao Capsule *v* Zhi Zhu Kuan Zhong Capsule | — | — | 1.39 (0.72, 2.54) | ⨁◯◯◯  Very low | 1.39 (0.72, 2.54) | ⨁◯◯◯  Very low |
| Xiao Pi Decoction *v* Zhi Zhu Kuan Zhong Capsule | — | — | 1.39 (0.62, 2.74) | ⨁◯◯◯  Very low | 1.39 (0.62, 2.74) | ⨁◯◯◯  Very low |
| He Wei Xiao Pi Decoction *v* Zhi Zhu Kuan Zhong Capsule | — | — | 1.32 (0.60, 2.57) | ⨁◯◯◯  Very low | 1.32 (0.60, 2.57) | ⨁◯◯◯  Very low |
| Tiao He Gan Pi Xing Qi Decoction *v* Zhi Zhu Kuan Zhong Capsule | — | — | 1.31 (0.59, 2.56) | ⨁◯◯◯  Very low | 1.31 (0.59, 2.56) | ⨁◯◯◯  Very low |
| Fu An Decoction *v* Zhi Zhu Kuan Zhong Capsule | — | — | 1.30 (0.59, 2.55) | ⨁◯◯◯  Very low | 1.30 (0.59, 2.55) | ⨁◯◯◯  Very low |
| Cai Zhu Jie Yu Decoction *v* Zhi Zhu Kuan Zhong Capsule | — | — | 1.29 (0.58, 2.51) | ⨁◯◯◯  Very low | 1.29 (0.58, 2.51) | ⨁◯◯◯  Very low |
| Domperidone *v* Zhi Zhu Kuan Zhong Capsule | 1.10 (0.96, 1.25) | ⨁◯◯◯  Very low^+‡^ | — | — | 1.13 (0.66, 1.84) | ⨁◯◯◯  Very low |
| Modified Zhi Zhu Decoction *v* Domperidone | **1.65 (1.31, 2.08)** | ⨁⨁◯◯  Low^‡^ | — | — | 1.69 (0.95, 2.80) | ⨁⨁◯◯  Low |
| Xiao Pi Tong Jiang Decoction *v* Domperidone | **1.35 (1.02, 1.79)** | ⨁⨁◯◯  Low^‡^ | — | — | 1.38 (0.76, 2.35) | ⨁⨁◯◯  Low |
| Xiao Pi Kuan Wei Decoction *v* Domperidone | **1.29 (1.07, 1.57)** | ⨁⨁◯◯  Low^‡^ | — | — | 1.33 (0.76, 2.18) | ⨁⨁◯◯  Low |
| Jian Pi Yi Qi Decoction *v* Domperidone | **1.29 (1.08, 1.55)** | ⨁⨁◯◯  Low^‡^ | — | — | 1.32 (0.76, 2.17) | ⨁⨁◯◯  Low |
| Shu Gan Jian Pi He Wei Decoction *v* Domperidone | **1.28 (1.06, 1.55)** | ⨁⨁◯◯  Low^‡^ | — | — | 1.31 (0.75, 2.16) | ⨁⨁◯◯  Low |
| Xiang Su Li Qi Decoction *v* Domperidone | 1.29 (0.99, 1.67) | ⨁⨁◯◯  Low^‡^ | — | — | 1.32 (0.74, 2.24) | ⨁⨁◯◯  Low |
| He Wei Decoction *v* Domperidone | **1.24 (1.09, 1.41)** | ⨁◯◯◯  Very low^+‡^ | — | — | 1.26 (0.86, 1.80) | ⨁◯◯◯  Very low |
| Modified He Gan Decoction *v* Domperidone | **1.23 (1.01, 1.51)** | ⨁⨁◯◯  Low^‡^ | — | — | 1.27 (0.72, 2.09) | ⨁⨁◯◯  Low |
| Liu Wei An Xiao Capsule *v* Domperidone | 1.23 (0.91, 1.65) | ⨁◯◯◯  Very low^+‡^ | — | — | 1.23 (0.85, 1.78) | ⨁◯◯◯  Very low |
| Xiao Pi Decoction *v* Domperidone | 1.19 (0.99, 1.44) | ⨁⨁◯◯  Low^‡^ | — | — | 1.23 (0.70, 2.03) | ⨁⨁◯◯  Low |
| He Wei Xiao Pi Decoction *v* Domperidone | 1.13 (0.98, 1.31) | ⨁◯◯◯  Very low^+‡^ | — | — | 1.17 (0.68, 1.90) | ⨁◯◯◯  Very low |
| Tiao He Gan Pi Xing Qi Decoction *v* Domperidone | 1.12 (0.94, 1.33) | ⨁⨁◯◯  Low^‡^ | — | — | 1.16 (0.67, 1.90) | ⨁⨁◯◯  Low |
| Fu An Decoction *v* Domperidone | 1.12 (0.95, 1.33) | ⨁⨁◯◯  Low^‡^ | — | — | 1.15 (0.66, 1.89) | ⨁⨁◯◯  Low |
| Cai Zhu Jie Yu Decoction *v* Domperidone | 1.11 (0.96, 1.28) | ⨁⨁◯◯  Low^‡^ | — | — | 1.14 (0.65, 1.85) | ⨁⨁◯◯  Low |
| Modified Zhi Zhu Decoction *v* Cai Zhu Jie Yu Decoction | — | — | 1.58 (0.70, 3.13) | ⨁⨁◯◯  Low | 1.58 (0.70, 3.13) | ⨁⨁◯◯  Low |
| Xiao Pi Tong Jiang Decoction *v* Cai Zhu Jie Yu Decoction | — | — | 1.29 (0.56, 2.60) | ⨁⨁◯◯  Low | 1.29 (0.56, 2.60) | ⨁⨁◯◯  Low |
| Xiao Pi Kuan Wei Decoction *v* Cai Zhu Jie Yu Decoction | — | — | 1.25 (0.56, 2.45) | ⨁⨁◯◯  Low | 1.25 (0.56, 2.45) | ⨁⨁◯◯  Low |
| Jian Pi Yi Qi Decoction *v* Cai Zhu Jie Yu Decoction | — | — | 1.24 (0.56, 2.44) | ⨁⨁◯◯  Low | 1.24 (0.56, 2.44) | ⨁⨁◯◯  Low |
| Shu Gan Jian Pi He Wei Decoction *v* Cai Zhu Jie Yu Decoction | — | — | 1.23 (0.55, 2.41) | ⨁⨁◯◯  Low | 1.23 (0.55, 2.41) | ⨁⨁◯◯  Low |
| Xiang Su Li Qi Decoction *v* Cai Zhu Jie Yu Decoction | — | — | 1.24 (0.55, 2.49) | ⨁⨁◯◯  Low | 1.24 (0.55, 2.49) | ⨁⨁◯◯  Low |
| He Wei Decoction *v* Cai Zhu Jie Yu Decoction | — | — | 1.18 (0.60, 2.14) | ⨁◯◯◯  Very low | 1.18 (0.60, 2.14) | ⨁◯◯◯  Very low |
| Modified He Gan Decoction *v* Cai Zhu Jie Yu Decoction | — | — | 1.19 (0.53, 2.34) | ⨁⨁◯◯  Low | 1.19 (0.53, 2.34) | ⨁⨁◯◯  Low |
| Liu Wei An Xiao Capsule *v* Cai Zhu Jie Yu Decoction | — | — | 1.15 (0.59, 2.12) | ⨁◯◯◯  Very low | 1.15 (0.59, 2.12) | ⨁◯◯◯  Very low |
| Xiao Pi Decoction *v* Cai Zhu Jie Yu Decoction | — | — | 1.15 (0.51, 2.27) | ⨁⨁◯◯  Low | 1.15 (0.51, 2.27) | ⨁⨁◯◯  Low |
| He Wei Xiao Pi Decoction *v* Cai Zhu Jie Yu Decoction | — | — | 1.09 (0.50, 2.13) | ⨁◯◯◯  Very low | 1.09 (0.50, 2.13) | ⨁◯◯◯  Very low |
| Tiao He Gan Pi Xing Qi Decoction *v* Cai Zhu Jie Yu Decoction | — | — | 1.08 (0.49, 2.13) | ⨁⨁◯◯  Low | 1.08 (0.49, 2.13) | ⨁⨁◯◯  Low |
| Fu An Decoction *v* Cai Zhu Jie Yu Decoction | — | — | 1.08 (0.48, 2.11) | ⨁⨁◯◯  Low | 1.08 (0.48, 2.11) | ⨁⨁◯◯  Low |
| Modified Zhi Zhu Decoction *v* Fu An Decoction | — | — | 1.56 (0.69, 3.10) | ⨁⨁◯◯  Low | 1.56 (0.69, 3.10) | ⨁⨁◯◯  Low |
| Xiao Pi Tong Jiang Decoction *v* Fu An Decoction | — | — | 1.28 (0.55, 2.58) | ⨁⨁◯◯  Low | 1.28 (0.55, 2.58) | ⨁⨁◯◯  Low |
| Xiao Pi Kuan Wei Decoction *v* Fu An Decoction | — | — | 1.23 (0.55, 2.43) | ⨁⨁◯◯  Low | 1.23 (0.55, 2.43) | ⨁⨁◯◯  Low |
| Jian Pi Yi Qi Decoction *v* Fu An Decoction | — | — | 1.22 (0.55, 2.41) | ⨁⨁◯◯  Low | 1.22 (0.55, 2.41) | ⨁⨁◯◯  Low |
| Shu Gan Jian Pi He Wei Decoction *v* Fu An Decoction | — | — | 1.21 (0.54, 2.39) | ⨁⨁◯◯  Low | 1.21 (0.54, 2.39) | ⨁⨁◯◯  Low |
| Xiang Su Li Qi Decoction *v* Fu An Decoction | — | — | 1.22 (0.53, 2.47) | ⨁⨁◯◯  Low | 1.22 (0.53, 2.47) | ⨁⨁◯◯  Low |
| He Wei Decoction *v* Fu An Decoction | — | — | 1.16 (0.58, 2.11) | ⨁◯◯◯  Very low | 1.16 (0.58, 2.11) | ⨁◯◯◯  Very low |
| Modified He Gan Decoction *v* Fu An Decoction | — | — | 1.17 (0.52, 2.32) | ⨁⨁◯◯  Low | 1.17 (0.52, 2.32) | ⨁⨁◯◯  Low |
| Liu Wei An Xiao Capsule *v* Fu An Decoction | — | — | 1.14 (0.58, 2.10) | ⨁◯◯◯  Very low | 1.14 (0.58, 2.10) | ⨁◯◯◯  Very low |
| Xiao Pi Decoction *v* Fu An Decoction | — | — | 1.13 (0.51, 2.25) | ⨁⨁◯◯  Low | 1.13 (0.51, 2.25) | ⨁⨁◯◯  Low |
| He Wei Xiao Pi Decoction *v* Fu An Decoction | — | — | 1.08 (0.48, 2.12) | ⨁◯◯◯  Very low | 1.08 (0.48, 2.12) | ⨁◯◯◯  Very low |
| Tiao He Gan Pi Xing Qi Decoction *v* Fu An Decoction | — | — | 1.07 (0.48, 2.10) | ⨁⨁◯◯  Low | 1.07 (0.48, 2.10) | ⨁⨁◯◯  Low |
| Modified Zhi Zhu Decoction *v* Tiao He Gan Pi Xing Qi Decoction | — | — | 1.55 (0.69, 3.09) | ⨁⨁◯◯  Low | 1.55 (0.69, 3.09) | ⨁⨁◯◯  Low |
| Xiao Pi Tong Jiang Decoction *v* Tiao He Gan Pi Xing Qi Decoction | — | — | 1.27 (0.55, 2.56) | ⨁⨁◯◯  Low | 1.27 (0.55, 2.56) | ⨁⨁◯◯  Low |
| Xiao Pi Kuan Wei Decoction *v* Tiao He Gan Pi Xing Qi Decoction | — | — | 1.23 (0.55, 2.41) | ⨁⨁◯◯  Low | 1.23 (0.55, 2.41) | ⨁⨁◯◯  Low |
| Jian Pi Yi Qi Decoction *v* Tiao He Gan Pi Xing Qi Decoction | — | — | 1.22 (0.54, 2.41) | ⨁⨁◯◯  Low | 1.22 (0.54, 2.41) | ⨁⨁◯◯  Low |
| Shu Gan Jian Pi He Wei Decoction *v* Tiao He Gan Pi Xing Qi Decoction | — | — | 1.21 (0.54, 2.39) | ⨁⨁◯◯  Low | 1.21 (0.54, 2.39) | ⨁⨁◯◯  Low |
| Xiang Su Li Qi Decoction *v* Tiao He Gan Pi Xing Qi Decoction | — | — | 1.22 (0.53, 2.44) | ⨁⨁◯◯  Low | 1.22 (0.53, 2.44) | ⨁⨁◯◯  Low |
| He Wei Decoction *v* Tiao He Gan Pi Xing Qi Decoction | — | — | 1.16 (0.58, 2.10) | ⨁◯◯◯  Very low | 1.16 (0.58, 2.10) | ⨁◯◯◯  Very low |
| Modified He Gan Decoction *v* Tiao He Gan Pi Xing Qi Decoction | — | — | 1.17 (0.52, 2.31) | ⨁⨁◯◯  Low | 1.17 (0.52, 2.31) | ⨁⨁◯◯  Low |
| Liu Wei An Xiao Capsule *v* Tiao He Gan Pi Xing Qi Decoction | — | — | 1.13 (0.58, 2.09) | ⨁◯◯◯  Very low | 1.13 (0.58, 2.09) | ⨁◯◯◯  Very low |
| Xiao Pi Decoction *v* Tiao He Gan Pi Xing Qi Decoction | — | — | 1.13 (0.50, 2.24) | ⨁⨁◯◯  Low | 1.13 (0.50, 2.24) | ⨁⨁◯◯  Low |
| He Wei Xiao Pi Decoction *v* Tiao He Gan Pi Xing Qi Decoction | — | — | 1.07 (0.48, 2.10) | ⨁◯◯◯  Very low | 1.07 (0.48, 2.10) | ⨁◯◯◯  Very low |
| Modified Zhi Zhu Decoction *v* He Wei Xiao Pi Decoction | — | — | 1.54 (0.69, 3.05) | ⨁◯◯◯  Very low | 1.54 (0.69, 3.05) | ⨁◯◯◯  Very low |
| Xiao Pi Tong Jiang Decoction *v* He Wei Xiao Pi Decoction | — | — | 1.26 (0.55, 2.52) | ⨁◯◯◯  Very low | 1.26 (0.55, 2.52) | ⨁◯◯◯  Very low |
| Xiao Pi Kuan Wei Decoction *v* He Wei Xiao Pi Decoction | — | — | 1.21 (0.55, 2.38) | ⨁◯◯◯  Very low | 1.21 (0.55, 2.38) | ⨁◯◯◯  Very low |
| Jian Pi Yi Qi Decoction *v* He Wei Xiao Pi Decoction | — | — | 1.20 (0.54, 2.37) | ⨁◯◯◯  Very low | 1.20 (0.54, 2.37) | ⨁◯◯◯  Very low |
| Shu Gan Jian Pi He Wei Decoction *v* He Wei Xiao Pi Decoction | — | — | 1.20 (0.54, 2.34) | ⨁◯◯◯  Very low | 1.20 (0.54, 2.34) | ⨁◯◯◯  Very low |
| Xiang Su Li Qi Decoction *v* He Wei Xiao Pi Decoction | — | — | 1.20 (0.53, 2.41) | ⨁◯◯◯  Very low | 1.20 (0.53, 2.41) | ⨁◯◯◯  Very low |
| He Wei Decoction *v* He Wei Xiao Pi Decoction | — | — | 1.15 (0.58, 2.07) | ⨁◯◯◯  Very low | 1.15 (0.58, 2.07) | ⨁◯◯◯  Very low |
| Modified He Gan Decoction *v* He Wei Xiao Pi Decoction | — | — | 1.15 (0.52, 2.27) | ⨁◯◯◯  Very low | 1.15 (0.52, 2.27) | ⨁◯◯◯  Very low |
| Liu Wei An Xiao Capsule *v* He Wei Xiao Pi Decoction | — | — | 1.12 (0.58, 2.06) | ⨁◯◯◯  Very low | 1.12 (0.58, 2.06) | ⨁◯◯◯  Very low |
| Xiao Pi Decoction *v* He Wei Xiao Pi Decoction | — | — | 1.12 (0.50, 2.21) | ⨁◯◯◯  Very low | 1.12 (0.50, 2.21) | ⨁◯◯◯  Very low |
| Modified Zhi Zhu Decoction *v* Xiao Pi Decoction | — | — | 1.47 (0.64, 2.92) | ⨁⨁◯◯  Low | 1.47 (0.64, 2.92) | ⨁⨁◯◯  Low |
| Xiao Pi Tong Jiang Decoction *v* Xiao Pi Decoction | — | — | 1.20 (0.52, 2.42) | ⨁⨁◯◯  Low | 1.20 (0.52, 2.42) | ⨁⨁◯◯  Low |
| Xiao Pi Kuan Wei Decoction *v* Xiao Pi Decoction | — | — | 1.16 (0.51, 2.28) | ⨁⨁◯◯  Low | 1.16 (0.51, 2.28) | ⨁⨁◯◯  Low |
| Jian Pi Yi Qi Decoction *v* Xiao Pi Decoction | — | — | 1.15 (0.51, 2.27) | ⨁⨁◯◯  Low | 1.15 (0.51, 2.27) | ⨁⨁◯◯  Low |
| Shu Gan Jian Pi He Wei Decoction *v* Xiao Pi Decoction | — | — | 1.14 (0.50, 2.26) | ⨁⨁◯◯  Low | 1.14 (0.50, 2.26) | ⨁⨁◯◯  Low |
| Xiang Su Li Qi Decoction *v* Xiao Pi Decoction | — | — | 1.15 (0.50, 2.32) | ⨁⨁◯◯  Low | 1.15 (0.50, 2.32) | ⨁⨁◯◯  Low |
| He Wei Decoction *v* Xiao Pi Decoction | — | — | 1.09 (0.55, 1.99) | ⨁◯◯◯  Very low | 1.09 (0.55, 1.99) | ⨁◯◯◯  Very low |
| Modified He Gan Decoction *v* Xiao Pi Decoction | — | — | 1.10 (0.48, 2.18) | ⨁⨁◯◯  Low | 1.10 (0.48, 2.18) | ⨁⨁◯◯  Low |
| Liu Wei An Xiao Capsule *v* Xiao Pi Decoction | — | — | 1.07 (0.54, 1.97) | ⨁◯◯◯  Very low | 1.07 (0.54, 1.97) | ⨁◯◯◯  Very low |
| Modified Zhi Zhu Decoction *v* Liu Wei An Xiao Capsule | — | — | 1.41 (0.69, 2.54) | ⨁◯◯◯  Very low | 1.41 (0.69, 2.54) | ⨁◯◯◯  Very low |
| Xiao Pi Tong Jiang Decoction *v* Liu Wei An Xiao Capsule | — | — | 1.16 (0.56, 2.13) | ⨁◯◯◯  Very low | 1.16 (0.56, 2.13) | ⨁◯◯◯  Very low |
| Xiao Pi Kuan Wei Decoction *v* Liu Wei An Xiao Capsule | — | — | 1.11 (0.55, 2.00) | ⨁◯◯◯  Very low | 1.11 (0.55, 2.00) | ⨁◯◯◯  Very low |
| Jian Pi Yi Qi Decoction *v* Liu Wei An Xiao Capsule | — | — | 1.11 (0.55, 1.98) | ⨁◯◯◯  Very low | 1.11 (0.55, 1.98) | ⨁◯◯◯  Very low |
| Shu Gan Jian Pi He Wei Decoction *v* Liu Wei An Xiao Capsule | — | — | 1.10 (0.54, 1.98) | ⨁◯◯◯  Very low | 1.10 (0.54, 1.98) | ⨁◯◯◯  Very low |
| Xiang Su Li Qi Decoction *v* Liu Wei An Xiao Capsule | — | — | 1.11 (0.53, 2.04) | ⨁◯◯◯  Very low | 1.11 (0.53, 2.04) | ⨁◯◯◯  Very low |
| He Wei Decoction *v* Liu Wei An Xiao Capsule | — | — | 1.05 (0.60, 1.71) | ⨁◯◯◯  Very low | 1.05 (0.60, 1.71) | ⨁◯◯◯  Very low |
| Modified He Gan Decoction *v* Liu Wei An Xiao Capsule | — | — | 1.06 (0.52, 1.91) | ⨁◯◯◯  Very low | 1.06 (0.52, 1.91) | ⨁◯◯◯  Very low |
| Modified Zhi Zhu Decoction *v* Modified He Gan Decoction | — | — | 1.42 (0.62, 2.83) | ⨁⨁◯◯  Low | 1.42 (0.62, 2.83) | ⨁⨁◯◯  Low |
| Xiao Pi Tong Jiang Decoction *v* Modified He Gan Decoction | — | — | 1.17 (0.50, 2.36) | ⨁⨁◯◯  Low | 1.17 (0.50, 2.36) | ⨁⨁◯◯  Low |
| Xiao Pi Kuan Wei Decoction *v* Modified He Gan Decoction | — | — | 1.12 (0.50, 2.23) | ⨁⨁◯◯  Low | 1.12 (0.50, 2.23) | ⨁⨁◯◯  Low |
| Jian Pi Yi Qi Decoction *v* Modified He Gan Decoction | — | — | 1.11 (0.50, 2.21) | ⨁⨁◯◯  Low | 1.11 (0.50, 2.21) | ⨁⨁◯◯  Low |
| Shu Gan Jian Pi He Wei Decoction *v* Modified He Gan Decoction | — | — | 1.11 (0.49, 2.20) | ⨁⨁◯◯  Low | 1.11 (0.49, 2.20) | ⨁⨁◯◯  Low |
| Xiang Su Li Qi Decoction *v* Modified He Gan Decoction | — | — | 1.11 (0.48, 2.24) | ⨁⨁◯◯  Low | 1.11 (0.48, 2.24) | ⨁⨁◯◯  Low |
| He Wei Decoction *v* Modified He Gan Decoction | — | — | 1.06 (0.53, 1.93) | ⨁◯◯◯  Very low | 1.06 (0.53, 1.93) | ⨁◯◯◯  Very low |
| Modified Zhi Zhu Decoction *v* He Wei Decoction | — | — | 1.39 (0.68, 2.54) | ⨁◯◯◯  Very low | 1.39 (0.68, 2.54) | ⨁◯◯◯  Very low |
| Xiao Pi Tong Jiang Decoction *v* He Wei Decoction | — | — | 1.13 (0.55, 2.11) | ⨁◯◯◯  Very low | 1.13 (0.55, 2.11) | ⨁◯◯◯  Very low |
| Xiao Pi Kuan Wei Decoction *v* He Wei Decoction | — | — | 1.09 (0.54, 1.97) | ⨁◯◯◯  Very low | 1.09 (0.54, 1.97) | ⨁◯◯◯  Very low |
| Jian Pi Yi Qi Decoction *v* He Wei Decoction | — | — | 1.08 (0.54, 1.97) | ⨁◯◯◯  Very low | 1.08 (0.54, 1.97) | ⨁◯◯◯  Very low |
| Shu Gan Jian Pi He Wei Decoction *v* He Wei Decoction | — | — | 1.08 (0.54, 1.95) | ⨁◯◯◯  Very low | 1.08 (0.54, 1.95) | ⨁◯◯◯  Very low |
| Xiang Su Li Qi Decoction *v* He Wei Decoction | — | — | 1.08 (0.53, 2.01) | ⨁◯◯◯  Very low | 1.08 (0.53, 2.01) | ⨁◯◯◯  Very low |
| Modified Zhi Zhu Decoction *v* Xiang Su Li Qi Decoction | — | — | 1.38 (0.59, 2.77) | ⨁⨁◯◯  Low | 1.38 (0.59, 2.77) | ⨁⨁◯◯  Low |
| Xiao Pi Tong Jiang Decoction *v* Xiang Su Li Qi Decoction | — | — | 1.13 (0.47, 2.30) | ⨁⨁◯◯  Low | 1.13 (0.47, 2.30) | ⨁⨁◯◯  Low |
| Xiao Pi Kuan Wei Decoction *v* Xiang Su Li Qi Decoction | — | — | 1.08 (0.47, 2.16) | ⨁⨁◯◯  Low | 1.08 (0.47, 2.16) | ⨁⨁◯◯  Low |
| Jian Pi Yi Qi Decoction *v* Xiang Su Li Qi Decoction | — | — | 1.08 (0.47, 2.15) | ⨁⨁◯◯  Low | 1.08 (0.47, 2.15) | ⨁⨁◯◯  Low |
| Shu Gan Jian Pi He Wei Decoction *v* Xiang Su Li Qi Decoction | — | — | 1.07 (0.46, 2.13) | ⨁⨁◯◯  Low | 1.07 (0.46, 2.13) | ⨁⨁◯◯  Low |
| Modified Zhi Zhu Decoction *v* Shu Gan Jian Pi He Wei Decoction | — | — | 1.37 (0.60, 2.73) | ⨁⨁◯◯  Low | 1.37 (0.60, 2.73) | ⨁⨁◯◯  Low |
| Xiao Pi Tong Jiang Decoction *v* Shu Gan Jian Pi He Wei Decoction | — | — | 1.12 (0.49, 2.27) | ⨁⨁◯◯  Low | 1.12 (0.49, 2.27) | ⨁⨁◯◯  Low |
| Xiao Pi Kuan Wei Decoction *v* Shu Gan Jian Pi He Wei Decoction | — | — | 1.08 (0.48, 2.15) | ⨁⨁◯◯  Low | 1.08 (0.48, 2.15) | ⨁⨁◯◯  Low |
| Jian Pi Yi Qi Decoction *v* Shu Gan Jian Pi He Wei Decoction | — | — | 1.07 (0.48, 2.13) | ⨁⨁◯◯  Low | 1.07 (0.48, 2.13) | ⨁⨁◯◯  Low |
| Modified Zhi Zhu Decoction *v* Jian Pi Yi Qi Decoction | — | — | 1.36 (0.60, 2.70) | ⨁⨁◯◯  Low | 1.36 (0.60, 2.70) | ⨁⨁◯◯  Low |
| Xiao Pi Tong Jiang Decoction *v* Jian Pi Yi Qi Decoction | — | — | 1.11 (0.48, 2.25) | ⨁⨁◯◯  Low | 1.11 (0.48, 2.25) | ⨁⨁◯◯  Low |
| Xiao Pi Kuan Wei Decoction *v* Jian Pi Yi Qi Decoction | — | — | 1.07 (0.48, 2.10) | ⨁⨁◯◯  Low | 1.07 (0.48, 2.10) | ⨁⨁◯◯  Low |
| Modified Zhi Zhu Decoction *v* Xiao Pi Kuan Wei Decoction | — | — | 1.35 (0.60, 2.69) | ⨁⨁◯◯  Low | 1.35 (0.60, 2.69) | ⨁⨁◯◯  Low |
| Xiao Pi Tong Jiang Decoction *v* Xiao Pi Kuan Wei Decoction | — | — | 1.11 (0.48, 2.23) | ⨁⨁◯◯  Low | 1.11 (0.48, 2.23) | ⨁⨁◯◯  Low |
| Modified Zhi Zhu Decoction *v* Xiao Pi Tong Jiang Decoction | — | — | 1.32 (0.56, 2.65) | ⨁⨁◯◯  Low | 1.32 (0.56, 2.65) | ⨁⨁◯◯  Low |

^@^The lower quality of evidence rating of the two direct comparisons constituted the quality of evidence rating of the indirect comparison.

^#^The quality of evidence rating of the direct or indirect comparison constituted the network’s quality of evidence rating.

^‡^Very serious imprecision: the total number of events was less than 300, and the upper or lower confidence limit crossed the minimally clinically important difference.

^+^Serious risk of bias: at least one study at high risk of bias was included.

**Appendix 5.** Effect estimates and quality of evidence ratings for comparisons in network meta-analysis on alleviating global symptoms at 2-week follow-up: domperidone as the comparator

| **Comparison** | **Direct evidence** | | **Indirect evidence** | | **Network meta-analysis** | |
| --- | --- | --- | --- | --- | --- | --- |
|  | Risk ratio (95% confidence interval) | Quality of evidence | Risk ratio (95% credible interval) | Quality of evidence^@^ | Risk ratio (95% credible interval) | Quality of evidence^#^ |
| Cai Hu Shu Gan Powder *v* Domperidone | **1.38 (1.08, 1.76)** | ⨁⨁◯◯  Low^‡^ | — | — | 1.41 (0.87, 2.20) | ⨁⨁◯◯  Low |
| Wei Kang Ping Decoction *v* Domperidone | **1.38 (1.08, 1.78)** | ⨁⨁◯◯  Low^‡^ | — | — | 1.40 (0.86, 2.18) | ⨁⨁◯◯  Low |
| Tiao Zhong Xiao Pi Decoction *v* Domperidone | 1.12 (0.98, 1.28) | ⨁⨁◯◯  Low^‡^ | — | — | 1.14 (0.74, 1.70) | ⨁⨁◯◯  Low |
| Wu Mo Decoction *v* Domperidone | 1.12 (0.95, 1.33) | ⨁⨁◯◯  Low^‡^ | — | — | 1.14 (0.73, 1.72) | ⨁⨁◯◯  Low |
| Ban Xia Xie Xin Decoction *v* Domperidone | 1.04 (0.86, 1.25) | ⨁⨁◯◯  Low^‡^ | — | — | 1.06 (0.66, 1.61) | ⨁⨁◯◯  Low |
| Qi Zhi Wei Tong Granules *v* Domperidone | 1.00 (0.92, 1.10) | ⨁⨁◯◯  Low^‡^ | — | — | 1.02 (0.67, 1.51) | ⨁⨁◯◯  Low |
| Cai Hu Shu Gan Powder *v* Qi Zhi Wei Tong Granules | — | — | 1.43 (0.74, 2.54) | ⨁⨁◯◯  Low | 1.43 (0.74, 2.54) | ⨁⨁◯◯  Low |
| Wei Kang Ping Decoction *v* Qi Zhi Wei Tong Granules | — | — | 1.42 (0.73, 2.52) | ⨁⨁◯◯  Low | 1.42 (0.73, 2.52) | ⨁⨁◯◯  Low |
| Tiao Zhong Xiao Pi Decoction *v* Qi Zhi Wei Tong Granules | — | — | 1.15 (0.62, 2.00) | ⨁⨁◯◯  Low | 1.15 (0.62, 2.00) | ⨁⨁◯◯  Low |
| Wu Mo Decoction *v* Qi Zhi Wei Tong Granules | — | — | 1.16 (0.61, 2.02) | ⨁⨁◯◯  Low | 1.16 (0.61, 2.02) | ⨁⨁◯◯  Low |
| Ban Xia Xie Xin Decoction *v* Qi Zhi Wei Tong Granules | — | — | 1.07 (0.57, 1.88) | ⨁⨁◯◯  Low | 1.07 (0.57, 1.88) | ⨁⨁◯◯  Low |
| Cai Hu Shu Gan Powder *v* Ban Xia Xie Xin Decoction | — | — | 1.39 (0.70, 2.52) | ⨁⨁◯◯  Low | 1.39 (0.70, 2.52) | ⨁⨁◯◯  Low |
| Wei Kang Ping Decoction *v* Ban Xia Xie Xin Decoction | — | — | 1.39 (0.70, 2.50) | ⨁⨁◯◯  Low | 1.39 (0.70, 2.50) | ⨁⨁◯◯  Low |
| Tiao Zhong Xiao Pi Decoction *v* Ban Xia Xie Xin Decoction | — | — | 1.13 (0.59, 1.98) | ⨁⨁◯◯  Low | 1.13 (0.59, 1.98) | ⨁⨁◯◯  Low |
| Wu Mo Decoction *v* Ban Xia Xie Xin Decoction | — | — | 1.13 (0.58, 1.99) | ⨁⨁◯◯  Low | 1.13 (0.58, 1.99) | ⨁⨁◯◯  Low |
| Cai Hu Shu Gan Powder *v* Wu Mo Decoction | — | — | 1.29 (0.65, 2.32) | ⨁⨁◯◯  Low | 1.29 (0.65, 2.32) | ⨁⨁◯◯  Low |
| Wei Kang Ping Decoction *v* Wu Mo Decoction | — | — | 1.28 (0.65, 2.31) | ⨁⨁◯◯  Low | 1.28 (0.65, 2.31) | ⨁⨁◯◯  Low |
| Tiao Zhong Xiao Pi Decoction *v* Wu Mo Decoction | — | — | 1.04 (0.55, 1.82) | ⨁⨁◯◯  Low | 1.04 (0.55, 1.82) | ⨁⨁◯◯  Low |
| Cai Hu Shu Gan Powder *v* Tiao Zhong Xiao Pi Decoction | — | — | 1.28 (0.66, 2.29) | ⨁⨁◯◯  Low | 1.28 (0.66, 2.29) | ⨁⨁◯◯  Low |
| Wei Kang Ping Decoction *v* Tiao Zhong Xiao Pi Decoction | — | — | 1.28 (0.66, 2.28) | ⨁⨁◯◯  Low | 1.28 (0.66, 2.28) | ⨁⨁◯◯  Low |
| Cai Hu Shu Gan Powder *v* Wei Kang Ping Decoction | — | — | 1.05 (0.52, 1.93) | ⨁⨁◯◯  Low | 1.05 (0.52, 1.93) | ⨁⨁◯◯  Low |

^@^The lower quality of evidence rating of the two direct comparisons constituted the quality of evidence rating of the indirect comparison.

^#^The quality of evidence rating of the direct or indirect comparison constituted the network’s quality of evidence rating.

^‡^Very serious imprecision: the total number of events was less than 300, and the upper or lower confidence limit crossed the minimally clinically important difference.

**Appendix 6.** Effect estimates and quality of evidence ratings for comparisons in network meta-analysis on alleviating global symptoms at 4-week follow-up: mosapride as the comparator

| **Comparison** | **Direct evidence** | | **Indirect evidence** | | **Network meta-analysis** | |
| --- | --- | --- | --- | --- | --- | --- |
|  | Risk ratio (95% confidence interval) | Quality of evidence | Risk ratio (95% credible interval) | Quality of evidence^@^ | Risk ratio (95% credible interval) | Quality of evidence^#^ |
| Da Li Tong Granules *v* Mosapride | **1.27 (1.01, 1.59)** | ⨁⨁◯◯  Low^‡^ | — | — | 1.28 (0.88, 1.83) | ⨁⨁◯◯  Low |
| Bu Gan Decoction *v* Mosapride | 1.04 (0.88, 1.23) | ⨁⨁◯◯  Low^‡^ | — | — | 1.05 (0.74, 1.46) | ⨁⨁◯◯  Low |
| Tiao Wei Xiao Pi Decoction *v* Mosapride | 1.02 (0.89, 1.16) | ⨁⨁◯◯  Low^‡^ | — | — | 1.03 (0.74, 1.40) | ⨁⨁◯◯  Low |
| Da Li Tong Granules *v* Tiao Wei Xiao Pi Decoction | — | — | 1.27 (0.77, 2.01) | ⨁⨁◯◯  Low | 1.27 (0.77, 2.01) | ⨁⨁◯◯  Low |
| Bu Gan Decoction *v* Tiao Wei Xiao Pi Decoction | — | — | 1.04 (0.64, 1.62) | ⨁⨁◯◯  Low | 1.04 (0.64, 1.62) | ⨁⨁◯◯  Low |
| Da Li Tong Granules *v* Bu Gan Decoction | — | — | 1.25 (0.74, 2.01) | ⨁⨁◯◯  Low | 1.25 (0.74, 2.01) | ⨁⨁◯◯  Low |

^@^The lower quality of evidence rating of the two direct comparisons constituted the quality of evidence rating of the indirect comparison.

^#^The quality of evidence rating of the direct or indirect comparison constituted the network’s quality of evidence rating.

^‡^Very serious imprecision: the total number of events was less than 300, and the upper or lower confidence limit crossed the minimally clinically important difference.

**Appendix 7.** Effect estimates and quality of evidence ratings for comparisons in network meta-analysis on alleviating postprandial fullness: domperidone as the comparator

| **Comparison** | **Direct evidence** | | **Indirect evidence** | | **Network meta-analysis** | |
| --- | --- | --- | --- | --- | --- | --- |
|  | Standardised mean difference (95% confidence interval) | Quality of evidence | Standardised mean difference (95% credible interval) | Quality of evidence^@^ | Standardised mean difference (95% credible interval) | Quality of evidence^#^ |
| Xiao Pi Kuan Wei Decoction v Domperidone | **-2.25 (-2.78, -1.73)** | ⨁⨁◯◯  Low^‡^ | — | — | -2.14 (-2.76, 0.70) | ⨁⨁◯◯  Low |
| Modified He Gan Decoction v Domperidone | **-0.71 (-1.17, -0.26)** | ⨁⨁◯◯  Low^‡^ | — | — | -0.39 (-1.55, 0.54) | ⨁⨁◯◯  Low |
| Modified Zhi Zhu Decoction v Domperidone | **-0.62 (-1.09, -0.15)** | ⨁⨁◯◯  Low^‡^ | — | — | -0.64 (-2.18, 0.74) | ⨁⨁◯◯  Low |
| He Wei Decoction v Domperidone | -0.47 (-1.23, 0.29) | ⨁◯◯◯  Very low^+‡^ | — | — | -0.12 (-1.68, 0.80) | ⨁◯◯◯  Very low |
| Xiao Pi Tong Jiang Decoction v Domperidone | -0.35 (-0.86, 0.16) | ⨁⨁◯◯  Low^‡^ | — | — | -0.34 (-2.28, 3.30) | ⨁⨁◯◯  Low |
| Xiao Pi Kuan Wei Decoction v Xiao Pi Tong Jiang Decoction | — | — | -2.09 (-3.51, 1.25) | ⨁⨁◯◯  Low | -2.09 (-3.51, 1.25) | ⨁⨁◯◯  Low |
| Modified He Gan Decoction v Xiao Pi Tong Jiang Decoction | — | — | -0.24 (-2.92, 1.60) | ⨁⨁◯◯  Low | -0.24 (-2.92, 1.60) | ⨁⨁◯◯  Low |
| Modified Zhi Zhu Decoction v Xiao Pi Tong Jiang Decoction | — | — | 0.31 (-5.25, 1.45) | ⨁⨁◯◯  Low | 0.31 (-5.25, 1.45) | ⨁⨁◯◯  Low |
| He Wei Decoction v Xiao Pi Tong Jiang Decoction | — | — | -0.28 (-3.94, 2.10) | ⨁◯◯◯  Very low | -0.28 (-3.94, 2.10) | ⨁◯◯◯  Very low |
| Xiao Pi Kuan Wei Decoction v He Wei Decoction | — | — | -2.12 (-2.99, 1.52) | ⨁◯◯◯  Very low | -2.12 (-2.99, 1.52) | ⨁◯◯◯  Very low |
| Modified He Gan Decoction v He Wei Decoction | — | — | -0.27 (-1.48, 1.55) | ⨁◯◯◯  Very low | -0.27 (-1.48, 1.55) | ⨁◯◯◯  Very low |
| Modified Zhi Zhu Decoction v He Wei Decoction | — | — | -0.54 (-1.37, 1.21) | ⨁◯◯◯  Very low | -0.54 (-1.37, 1.21) | ⨁◯◯◯  Very low |
| Xiao Pi Kuan Wei Decoction v Modified Zhi Zhu Decoction | — | — | -2.03 (-3.01, 2.88) | ⨁⨁◯◯  Low | -2.03 (-3.01, 2.88) | ⨁⨁◯◯  Low |
| Modified He Gan Decoction v Modified Zhi Zhu Decoction | — | — | -0.04 (-0.94, 2.53) | ⨁⨁◯◯  Low | -0.04 (-0.94, 2.53) | ⨁⨁◯◯  Low |
| Xiao Pi Kuan Wei Decoction v Modified He Gan Decoction | — | — | -1.76 (-2.52, 0.37) | ⨁⨁◯◯  Low | -1.76 (-2.52, 0.37) | ⨁⨁◯◯  Low |

^@^The lower quality of evidence rating of the two direct comparisons constituted the quality of evidence rating of the indirect comparison.

^#^The quality of evidence rating of the direct or indirect comparison constituted the network’s quality of evidence rating.

^‡^Very serious imprecision: the total number of participants was less than 400, and the upper or lower confidence limit crossed the minimally clinically important difference.

^+^Serious risk of bias: at least one study at high risk of bias was included.

**Appendix 8.** Effect estimates and quality of evidence ratings for comparisons in network meta-analysis on alleviating early satiety: domperidone as the comparator

| **Comparison** | **Direct evidence** | | **Indirect evidence** | | **Network meta-analysis** | |
| --- | --- | --- | --- | --- | --- | --- |
|  | Standardised mean difference (95% confidence interval) | Quality of evidence | Standardised mean difference (95% credible interval) | Quality of evidence^@^ | Standardised mean difference (95% credible interval) | Quality of evidence^#^ |
| Xiao Pi Kuan Wei Decoction *v* Domperidone | **-3.48 (-4.13, -2.83)** | ⨁⨁◯◯  Low^‡^ | — | — | **-3.90 (-0.68, -0.42)** | ⨁⨁◯◯  Low |
| He Wei Decoction *v* Domperidone | **-0.51 (-0.81, -0.20)** | ⨁◯◯◯  Very low^+‡^ | — | — | -0.58 (-2.69, 2.06) | ⨁⨁◯◯  Low |
| Xiao Pi Tong Jiang Decoction *v* Domperidone | -0.35 (-0.86, 0.16) | ⨁⨁◯◯  Low^‡^ | — | — | -0.04 (-2.12, 1.46) | ⨁◯◯◯  Very low |
| Xiao Pi Kuan Wei Decoction *v* Xiao Pi Tong Jiang Decoction | **—** | **—** | -3.27 (-6.34, 0.24) | ⨁⨁◯◯  Low | -3.27 (-6.34, 0.24) | ⨁⨁◯◯  Low |
| He Wei Decoction *v* Xiao Pi Tong Jiang Decoction | — | — | -0.49 (-4.38, 1.84) | ⨁◯◯◯  Very low | -0.49 (-4.38, 1.84) | ⨁◯◯◯  Very low |
| Xiao Pi Kuan Wei Decoction *v* He Wei Decoction | — | — | -3.27 (-4.81, 1.95) | ⨁◯◯◯  Very low | -3.27 (-4.81, 1.95) | ⨁◯◯◯  Very low |

^@^The lower quality of evidence rating of the two direct comparisons constituted the quality of evidence rating of the indirect comparison.

^#^The quality of evidence rating of the direct or indirect comparison constituted the network’s quality of evidence rating.

^‡^Very serious imprecision: the total number of participants was less than 400, and the upper or lower confidence limit crossed the minimally clinically important difference.

^+^Serious risk of bias: at least one study at high risk of bias was included.

**Appendix 9.** Effect estimates and quality of evidence ratings for comparisons in network meta-analysis on alleviating epigastric pain: domperidone as the comparator

| **Comparison** | **Direct evidence** | | **Indirect evidence** | | **Network meta-analysis** | |
| --- | --- | --- | --- | --- | --- | --- |
|  | Standardised mean difference (95% confidence interval) | Quality of evidence | Standardised mean difference (95% credible interval) | Quality of evidence^@^ | Standardised mean difference (95% credible interval) | Quality of evidence^#^ |
| Xiao Pi Kuan Wei Decoction *v* Domperidone | **-1.19 (-1.63, -0.75)** | ⨁⨁◯◯  Low^‡^ | **—** | **—** | **-1.23 (-1.66, -0.29)** | ⨁⨁◯◯  Low |
| He Wei Decoction *v* Domperidone | **-0.78 (-1.10, -0.47)** | ⨁◯◯◯  Very low^+‡^ | **—** | **—** | **-0.64 (-1.18, -0.33)** | ⨁◯◯◯  Very low |
| Xiao Pi Tong Jiang Decoction *v* Domperidone | **-0.53 (-1.05, -0.02)** | ⨁⨁◯◯  Low^‡^ | **—** | **—** | **-0.70 (-1.44, -0.35)** | ⨁⨁◯◯  Low |
| Xiao Pi Kuan Wei Decoction *v* Xiao Pi Tong Jiang Decoction | **—** | **—** | -0.23 (-1.14, 0.36) | ⨁⨁◯◯  Low | -0.23 (-1.14, 0.36) | ⨁⨁◯◯  Low |
| He Wei Decoction *v* Xiao Pi Tong Jiang Decoction | **—** | **—** | 0.01 (-0.77, 1.03) | ⨁◯◯◯  Very low | 0.01 (-0.77, 1.03) | ⨁◯◯◯  Very low |
| Xiao Pi Kuan Wei Decoction *v* He Wei Decoction | **—** | **—** | -0.53 (-1.30, 0.65) | ⨁◯◯◯  Very low | -0.53 (-1.30, 0.65) | ⨁◯◯◯  Very low |

^@^The lower quality of evidence rating of the two direct comparisons constituted the quality of evidence rating of the indirect comparison.

^#^The quality of evidence rating of the direct or indirect comparison constituted the network’s quality of evidence rating.

^‡^Very serious imprecision: the total number of participants was less than 400, and the upper or lower confidence limit crossed the minimally clinically important difference.

^+^Serious risk of bias: at least one study at high risk of bias was included.
